# Formal synthesis of a selective estrogen receptor modulator with tetrahydrofluorenone structure using [3 + 2 + 1] cycloaddition of yne-vinylcyclopropanes and CO

**DOI:** 10.3762/bjoc.21.127

**Published:** 2025-08-14

**Authors:** Jing Zhang, Guanyu Zhang, Hongxi Bai, Zhi-Xiang Yu

**Affiliations:** 1 Pingshan Translational Medicine Center, Shenzhen Bay Laboratory, Shenzhen, 518118, China; 2 Beijing National Laboratory of Molecular Sciences (BNLMS), Key Laboratory of Bioorganic Chemistry and Molecular Engineering, College of Chemistry, Peking University, Beijing, 100871, China

**Keywords:** [3 + 2 + 1] cycloaddition, selective estrogen receptor modulators, synthesis, tetrahydrofluorenone

## Abstract

A formal synthesis of product **VI** with tetrahydroflurenone structure as selective estrogen receptor modulator has been realized. The Rh-catalyzed [3 + 2 + 1] reaction of yne-vinylcyclopropanes and CO (20 mmol scale, in 87% yield) for building the 6/5/5 skeleton, and a Heck coupling reaction constructing the [3.2.1] framework, are the two key reactions in this 11-step synthesis.

## Introduction

Estrogen receptors (ERs) [1–2] are widely distributed nuclear receptor proteins and include two subtypes, ERα [3–4] and ERβ [5–6]. These receptors can bind 17β-estradiol with similar affinity, facilitating the transfer of estrogen to various tissues in the body. Due to this, 17β-estradiol as non-selective ligand has been extensively studied in hormone replacement therapy (HRT). However, HRT produced some risks of breast and uterine cancer. Consequently, scientists then concentrated their efforts on developing selective estrogen receptor modulators (SERMs) that interact with intracellular ERs in a tissue-specific manner to reduce the risk of estrogen-related cancers and other complications. Now there is a growing consensus that specific ERβ agonists are safer than nonspecific modulators by avoiding ERα stimulation [7–9]. Therefore, searching for SERMs toward ERβ as agonist and/or antagonist [10–11] has become a research frontier for treating breast cancer, osteoporosis, cardiovascular disease, neuropathies, and other diseases.

**Scheme 1:**
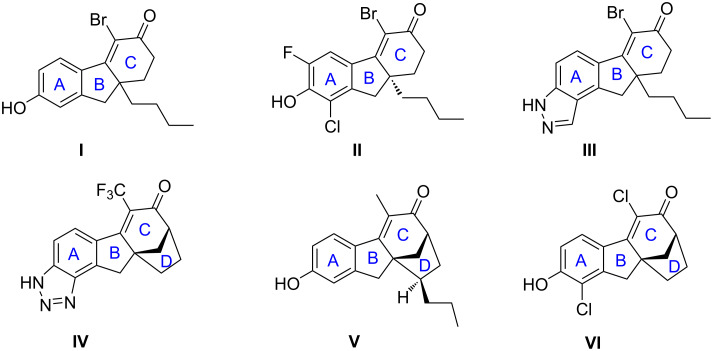
Reported biologically active tetrahydrofluorenone-SERMs molecules.

Merck scientists found that molecules with tetrahydrofluorenone (6/5/6 tricyclic motif) can act as SERMs. For example, molecules **I** and **II** (Scheme 1A) displayed low nanomolar affinity for ERβ and have 75-fold selectivity of ERβ over Erα [12]. Molecules **III** and **IV** (Scheme 1A) with an additional pyrazole ring compared to **I** and **II** had good pharmacokinetic properties that had overcome the problems of rapid clearance and low oral bioavailability executed by previous molecules [13–14]. A series of bridged tetrahydrofluorenone derivatives, represented by molecules **V** and **VI,** showed significant ERβ binding affinity and high selectivity [15–19].

**Scheme 2:**
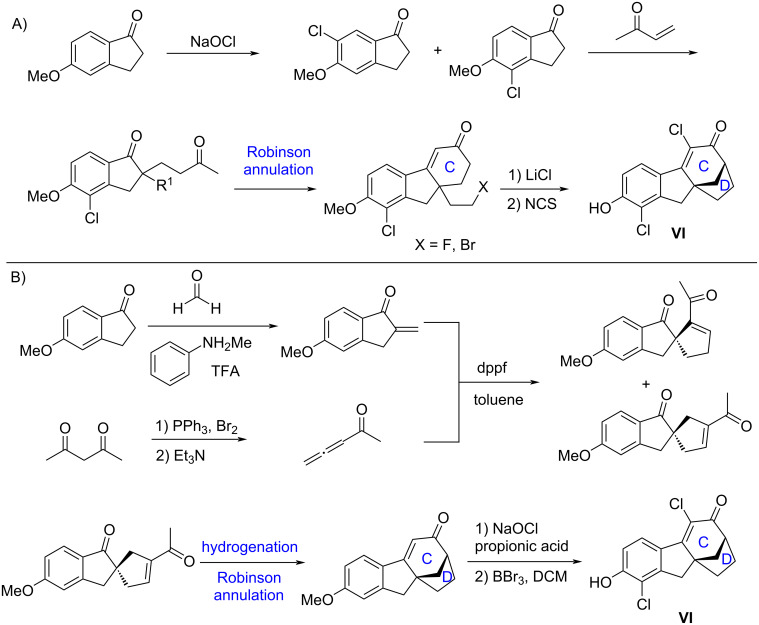
Reported synthesis routes to SERMs molecule **VI**.

So far, there are only two routes for accessing bridged tetrahydrofluorenone derivative **VI**. The first one shown in Scheme 2A includes a Robinson annulation to construct the **C** ring (cyclohexenone ring), and an intramolecular S_N_2 reaction to build the D ring (five-membered ring) [15–18]. In 2013, Wallace and co-workers disclosed the second route for this molecule (Scheme 2B) [19], in which the five-membered ring B was formed by utilizing asymmetric Lu [3 + 2] cycloaddition reaction [20–21] between indanone and allenyl ketone. Then hydrogenation and Robinson annulation delivered the core of the target molecule. Some other excellent synthetic routes for tetrahydrofluorenone derivates have been developed [12–19] but finding new strategies for these molecules and their derivatives are still required for future medicinal investigations. Due to this, we decided to explore a new approach to **VI**, which is reported here.

**Scheme 3:**
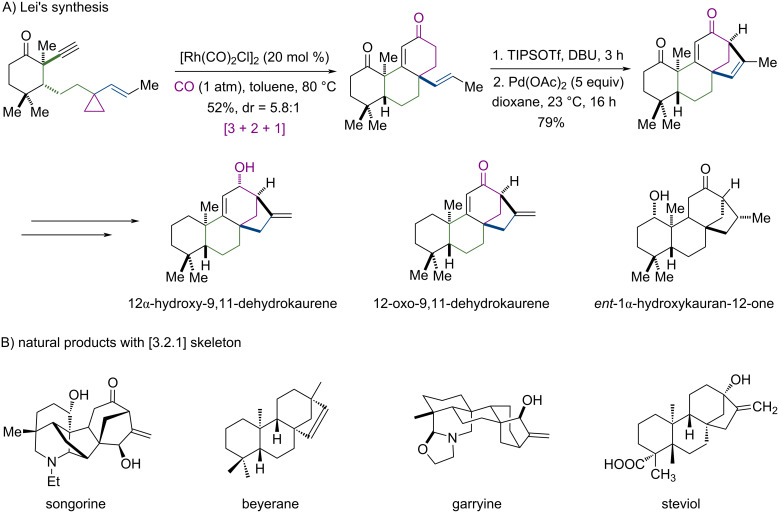
Lei’s synthesis of natural products of ent-kaurane diterpenoids (A), and natural products songorine, beyerene, garryine and steviol (B).

Our approach is inspired by the Lei’s synthesis of ent-kaurane diterpenoids (Scheme 3A) [22] which share the [3.2.1] motif as the SERMs in Scheme 1 do. Lei used a [3 + 2 + 1] reaction [23–28], which was developed in our group and has been applied in synthesis, coupled with stoichiometric Pd-mediated Heck reaction, concisely reaching the framework of their target natural products. We decided to use the same approach to synthesize **VI**, but we planned to use a catalytic Heck reaction by using a stronger nucleophile. As can be seen below, stoichiometric Heck reaction for **VI** failed because the present vinyl group of the [3 + 2 + 1] product does not have a methyl group in the terminal position, which could be the key to Lei’s synthesis. Realizing the synthesis of **VI** would provide a practical strategy not only to our target here but also to other natural products with [3.2.1] framework such as songorine, beyerene, garryine and steviol shown in Scheme 3B.

## Results and Discussion

**Scheme 4:**
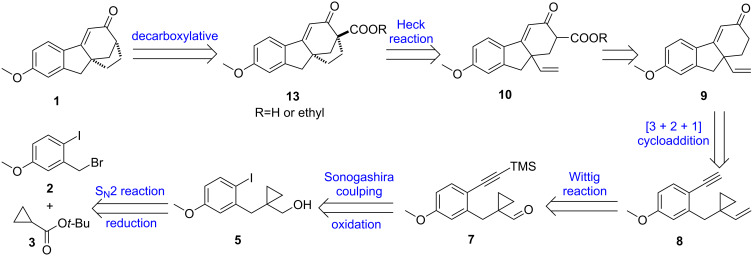
Retrosynthetic analysis for the synthesis of **1**.

Scheme 4 is the retrosynthetic analysis for the key intermediate **1**, which can reach the final compound **VI** via chlorination and demethylation [19]. Target molecule **1** can be accessed by decarboxylation reaction from compound **13**, prepared by an intramolecular Heck reaction between the β-ketoester and the vinyl group in compound **10**. Compound **10** can be realized by introducing an ester group in **9**, which is the [3 + 2 + 1] cycloadduct from **8** and CO using a Rh catalyst. The [3 + 2 + 1] substrate of yne-vinylcyclopropane (yne-VCP) **8** can be synthesized by Wittig reaction from cyclopropyl aldehyde **7**, in which the alkyne moiety is installed via Sonogashira coupling reaction using aryl iodide **5**. The cyclopropyl ring in **5** can be introduced via an S_N_2 reaction of compound **2** with *tert*-butyl cyclopropanecarboxylate (**3**).

**Scheme 5:**
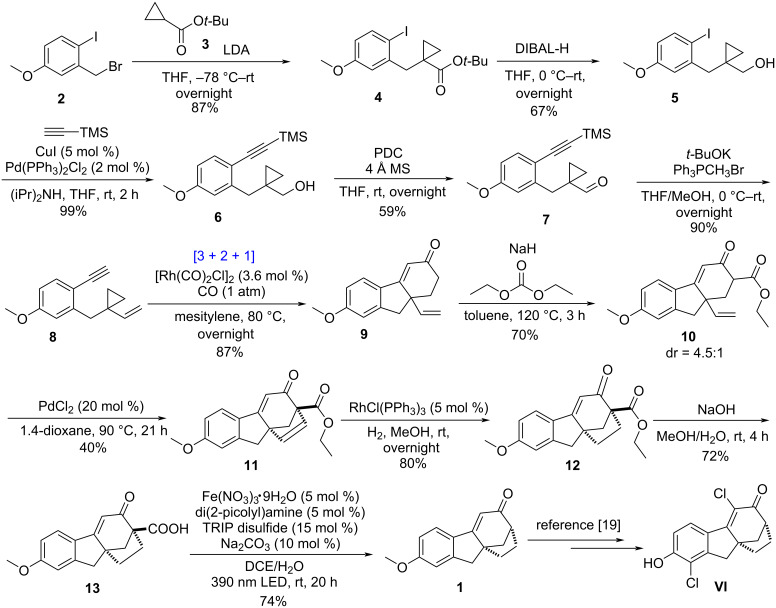
Formal synthesis of SERMs molecule **VI**.

Scheme 5 summarizes the final successful execution of this route. The starting material **2** is a known compound [29] and can be prepared from readily available *m*-anisyl alcohol by using iodination and bromination reactions (see Supporting Information File 1 for the details). Subsequently, an S_N_2 reaction between **2** and *tert*-butyl cyclopropanecarboxylate (**3**) in the presence of LDA delivered product **4** in 87% yield with a cyclopropyl group. Then reducing the carboxylate group in **4** with DIBAL-H afforded alcohol **5** in 67% yield. Next, Sonogashira coupling reaction between **5** and trimethylsilylacetylene generated **6** with an alkyne moiety quantitatively. After that, the hydroxy group in **6** was oxidized into a carbonyl group, giving **7** in 59% yield. Then, under basic conditions, the aldehyde group in **7** was converted into a vinyl group via Wittig reaction, affording yne-VCP substrate **8** in 90% yield. During this process, the TMS protecting group was lost.

We then investigated the [3 + 2 + 1] reaction of **8** and CO. Applying the traditional solvent dioxane for the [Rh(CO)_2_Cl]_2_ catalyzed [3 + 2 + 1] reaction (the catalyst loading was increased from 5 mol % to 10 mol %) gave **9** in only 26% yield. To our delight, the reaction yield could be improved to 87% by using mesitylene [30] as the solvent and the loading of [Rh(CO)_2_Cl]_2_ catalyst can be reduced to 3.6 mol % (the reaction scale was 20.6 mmol).

After finishing the key [3 + 2 + 1] reaction, we focused on building the D ring in **1**. Initially, we tried to directly close the ring through addition of the α position of the carbonyl group to the bridgehead vinyl group through Heck reaction (using Pd catalyst), but disappointingly, all efforts failed to realize this goal. A stoichiometric version of the Heck reaction used by Lei did not work either. Maybe the terminal vinyl group in **9** has a lower reactivity compared to Lei’s substrate (Scheme 3). We then decided to introduce an ester group at the α position of the carbonyl group in **9** to get compound **10**, which could have a more nucleophilic carbon better for the Heck reaction. **10** was obtained in 70% yield with a diastereomeric ratio of 4.5:1. Then, we screened various palladium catalysts and solvents to accomplish the target Heck coupling, finding that using 1,4-dioxane as the solvent and 20 mol % PdCl_2_ as the catalyst, **11** could be obtained in 40% yield in the air. We tried by using more catalyst, or adding O_2_ (or CuCl_2_) as oxidant, but all these efforts did not give improved reaction yields (the reason for this was not known).

A hydrogenation reaction to reduce the C=C bond in **11** was then successfully applied, delivering product **12** in 80% yield (5 mol % RhCl(PPh_3_)_3_ catalyst and 1 atm hydrogen atmosphere were used). Next, we tested whether Krapcho decarboxylation reaction can convert **12** into **1** in one step. Unfortunately, failure was encountered here. This can be expected because the reaction site here is a bridge quaternary center (no such example was reported in literature for this) [31–33]. Due to this, we then converted the ester group in **12** into a carboxylic acid group, reaching **13** in 72% yield. Finally, photocatalytic decarboxylation [34] delivered the desired product **1** in 74% yield, realizing a formal synthesis of the selective estrogen receptor modulator **VI**.

## Conclusion

In conclusion, we achieved a formal synthesis of SERM molecule VI through a 11-step process to its precursor, molecule **1**. Two key reactions have been applied here. The first one is a [3 + 2 + 1] reaction of yne-VCP and CO to build the 6/5/6 skeleton in 20 mmol scale with 87% yield. The second one is a Heck reaction between the β-ketoester and the vinyl group (coming from the [3 + 2 + 1] reaction) to form the [3.2.1] ring, the D ring of the target molecule. This route can provide new derivatives for further searching new SERMs. The synthetic strategy can be applied to other molecules with [3.2.1] framework. Of the same importance, the gram scale (4 g) of the [3 + 2 + 1] reaction with 87% reaction yield demonstrates the practical use of this reaction in synthesis.

## Supporting Information

File 1 Experimental procedures, product characterizations, and copies of the ^1^H and ^13^C NMR spectra.

## Data Availability

All data that supports the findings of this study is available in the published article and/or the supporting information of this article.
