# Supplementary material for: Formal synthesis of a selective estrogen receptor modulator with tetrahydrofluorenone structure using [3 + 2 + 1] cycloaddition of yne-vinylcyclopropanes and CO
Source: Beilstein J Org Chem. 2025 Aug 14;21:1639–44. doi: 10.3762/bjoc.21.127 (PMC12362305; doi:10.3762/bjoc.21.127)
Supplement: File 1 — Experimental procedures, product characterizations, and copies of the 1H and 13C NMR spectra. [file Beilstein_J_Org_Chem-21-1639-s001.pdf]

BEILSTEIN JOURNAL OF ORGANIC CHEMISTRY

Supporting Information

for

Formal synthesis of a selective estrogen receptor modulator with tetrahydrofluorenone structure using [3 + 2 + 1] cycloaddition of yne-vinylcyclopropanes and CO

Jing Zhang, Guanyu Zhang, Hongxi Bai and Zhi-Xiang Yu

Beilstein J. Org. Chem. **2025,** *21,* 1639–1644. doi:10.3762/bjoc.21.127

Experimental procedures, product characterizations, and copies of the ^{1}H and ^{13}C NMR spectra

License and Terms: This is a supporting information file under the terms of the Creative Commons Attribution License (https://creativecommons.org/licenses/by/4.0). Please note that the reuse, redistribution and reproduction in particular requires that the author(s) and source are credited and that individual graphics may be subject to special legal provisions.
The license is subject to the *Beilstein Journal of Organic Chemistry* terms and conditions: (https://www.beilstein-journals.org/bjoc/terms)

Table of contents

1.General information S3

2.Total synthesis of selective estrogen receptor modulator S3

3. References. S14

4. NMR spectra of products **1–15** S15

1.General information

Unless otherwise noted, materials were purchased from commercial suppliers (Alfa, TCI and Sigma-Aldrich etc.), and used without further purification. All the solvents were treated according to general methods. All reactions were monitored by thin-layer chromatography (TLC) on silica gel plates using UV light as visualizing agent (if applicable). Flash column chromatography was performed using 200–300 mesh silica gel. Nuclear magnetic resonance (NMR) spectra were measured on Bruker AVANCE NEO 400 (^{1}H at 400 MHz, ^{13}C at 101 MHz). Data for ^{1}H NMR spectrum are reported as follows: chemical shift δ (ppm) referenced to tetramethylsilane (TMS, 0.00 ppm), $CDCl_3$ (7.26 ppm). Data are reported as follows: chemical shift, multiplicity (s = singlet, brs = broad singlet, d = doublet, t = triplet, q = quartet, dd = doublet of doublets, dt = doublet of triplets, dq = doublet of quartets, tt = triplet of triplets, ddd = doublet of doublet of doublets, ddt = doublet of doublet of triplets, m = multiplet), coupling constant *J* (Hz), and integration. Data for ^{13}C NMR spectrum are reported as follows: chemical shift δ (ppm) referenced to $CDCl_3$ (77.00 ppm). The high-resolution mass spectra (HRMS) were measured on a Shimadzu LCMS-IT-TOF mass spectrometer or DIONEX UltiMate 3000 & Bruker Compact TOF mass spectrometer by ESI and Thermo fisher Q-Exactive mass spectrometer by ACPI. Measured values are reported to 4 decimal places of the calculated value. The calculated values are based on the most abundant isotope.

2.Total synthesis of selective estrogen receptor modulator

Scheme S1. Total synthesis

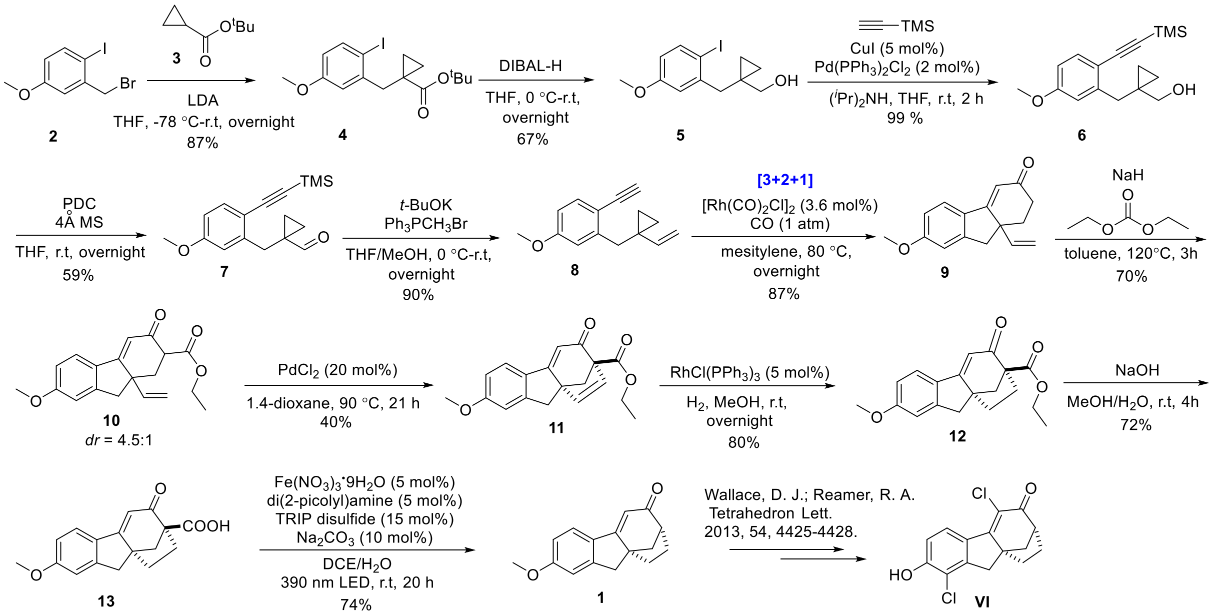

Synthesis of 2[1]

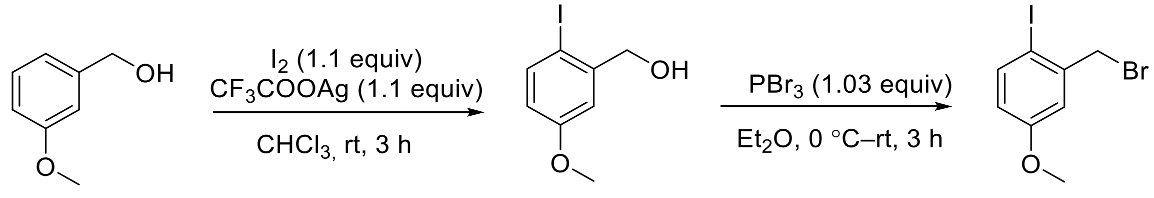

SI S2 2

Preparation of S1 (known compound): A solution of I_2 (1.1 equiv) in $CHCl_3$ (0.4 M) was added over a suspension CF_3COOAg (1.1 equiv) and substrate **S1** (1.0 equiv) in $CHCl_3$(0.25 M). The reaction mixture was stirred at rt for 3 h. The resulting AgI precipitate was filtered, and the resulting solution was washed with saturated $Na_2S_2O_3$ then extracted with EA. The combined organic layers were washed with brine, dried over anhydrous Na_2SO_4, filtered, and concentrated by rotary evaporation. Without further purification, the crude product is used directly in the next step.

Preparation of S2 (known compound): To a solution of **S2** (1.0 equiv) in Et_2O (0.25 M) was added PBr_3 (1.03 equiv) by syringe at 0 °C. Completed the addition, the reaction mixture was warmed to room temperature and stirred for 3 h. Quenched with water at 0 °C, saturated $NaHCO_3$ solution was added until no bubbles were produced, extracted with dichloromethane. The combined organic layers were washed with brine, dried over anhydrous Na_2SO_4, filtered, and concentrated by rotary evaporation without further purification to attain the target product **2** as a white solid.

Synthesis of 4

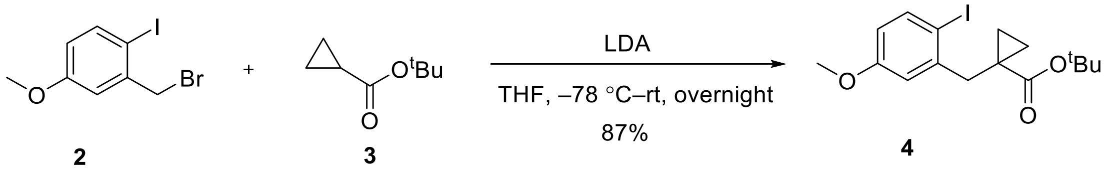

2 3 4

To a flask added $(iPr)_2NH$ (0.211 mL, 1.5 mmol, 1.18 equiv), *n*-BuLi (1.6 M, 1.1 mL, 1.78 mmol, 1.4 equiv), and dry THF (4 mL) at −78 °C under argon and stirred for 30 min. Then **3** *tert*-butyl cyclopropanecarboxylate (0.27 mL, 1.91 mmol, 1.5 equiv) was added slowly by syringe and stirred at −78 °C for 4 h. After that, **2** (413.7 mg, 1.27

mmol, 1.0 equiv) in dry THF (2.35 mL) was added to the above reaction mixture. This reaction mixture was then warmed to room temperature and stirred overnight. Finally, the reaction was quenched with saturated NH_4Cl, extracted with EA. The combined organic layers were washed with brine, dried over anhydrous Na_2SO_4, filtered, and concentrated by rotary evaporation. Purification of the crude product by flash column chromatography (silica gel, 100:1–50:1 petroleum ether/EtOAc) afforded the target compound **4** (430.2 mg, 87%) as colorless oil.

TLC (30:1 PE/EA, R_f): 0.4

^{1}H NMR (400 MHz, CDCl$_3$) δ = 7.67 (d, J = 8.6 Hz, 1H), 6.95 (d, J = 3.0 Hz, 1H), 6.51 (dd, J = 8.7, 3.0 Hz, 1H), 3.76 (s, 3H), 2.99 (s, 2H), 1.35 (s, 9H), 1.30 (q, J = 4.1 Hz, 2H), 0.75 (q, J = 4.1 Hz, 2H). **^{13}C NMR (101 MHz, CDCl$_3$)** δ = 174.0, 159.9, 143.7, 139.6, 115.2, 113.8, 90.5, 80.5, 55.4, 43.5, 28.1, 23.4, 15.1.

HRMS (ESI): m/z $[M + Na]^+$ calcd for $C_{16}H_{21}IO_3Na^+$: 411.0428; found: 411.0436.

Synthesis of 5

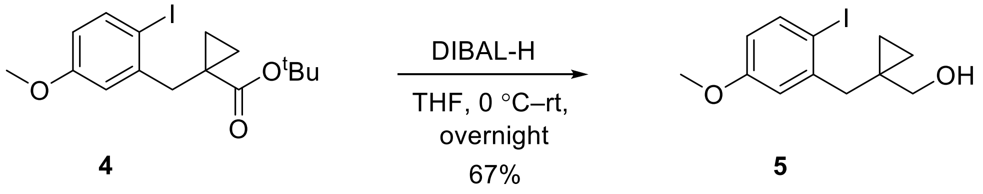

To a solution of **4** (430.2 mg, 1.1 mmol, 1.0 equiv) in DCM (0.25M, 4.4 mL) was added DIBAL-H (1.5 M, 2.2 mL, 3.3 mmol, 3.0 equiv) by syringe at 0 °C under argon. Then the reaction mixture warmed to room temperature and stirred overnight. The reaction mixture was then quenched slowly with saturated seignette salt ($KNaC_4H_4O_6{\cdot}4H_2O$) at 0 °C, added Na_2SO_4 and stirred at rt for 1 h, filtered and concentrated by rotary evaporation. Purification of the crude product by flash column chromatography (silica gel, 10:1–5:1 petroleum ether/EtOAc) afforded the target compound **5** (233.9 mg, 67%) as a colorless oil.

TLC (3:1 PE/EA, R_f): 0.4

^{1}H NMR (400 MHz, CDCl$_3$) δ = 7.68 (d, *J* = 8.7 Hz, 1H), 6.89 (d, *J* = 3.0 Hz, 1H), 6.52 (dd, *J* = 8.7, 3.1 Hz, 1H), 3.77 (s, 3H), 3.44 (s, 2H), 2.95 (s, 2H), 1.57 (s, 1H), 0.48 (q, *J* = 2.0 Hz, 4H).. **^{13}C NMR (101 MHz, CDCl$_3$)** δ = 159.7, 143.1, 139.7, 116.5, 113.8, 90.8, 69.1, 55.3, 42.8, 22.5, 9.1.

HRMS (ESI): m/z [M]$^+$ calcd for $C_{12}H_{15}IO_2^+$. 318.0111; found: 318.0107.

Synthesis of 6

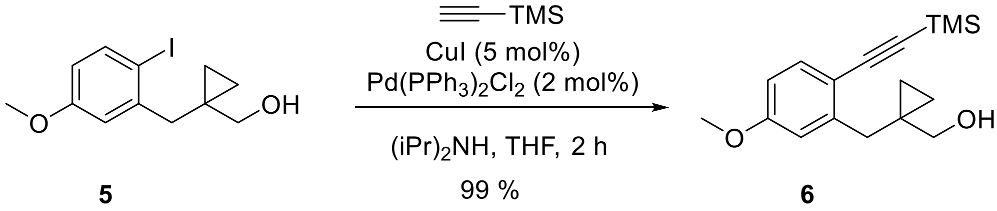

To a solution of CuI (142.8 mg, 0.75 mmol, 0.05 equiv) and $Pd(PPh_3)_2Cl_2$ (210.6 mg, 0.3 mmol, 0.02 equiv) in $(iPr)_2NH$ (88 mL) was added **5** (4.7702 g, 15 mmol, 1.0 equiv) in THF (88 mL) at rt under argon. Then trimethylsilylacetylene (6.36 mL, 45 mmol, 3.0 equiv) was added to the above reaction mixture, during which, color of the reaction mixture changed from orange to black. After that, the reaction mixture was stirred at rt for 2 h. Then the solvent was removed by rotary evaporation. Purification of the crude product by flash column chromatography (silica gel, 10:1–5:1 petroleum ether/EtOAc) afforded the target compound **6** (4.72 g, 99%) as colorless oil.

TLC (3:1 PE/EA, R*f*): 0.4

^{1}H NMR (400 MHz, CDCl$_3$) δ = 7.41 (d, *J* = 8.5 Hz, 1H), 6.79 (d, *J* = 2.6 Hz, 1H), 6.71 (dd, *J* = 8.5, 2.6 Hz, 1H), 3.80 (s, 3H), 3.29 (d, *J* = 5.5 Hz, 2H), 2.90 (s, 2H), 0.62 – 0.58 (m, 2H), 0.47 – 0.43 (m, 2H), 0.26 (s, 9H). **^{13}C NMR (101 MHz, CDCl$_3$)** δ = 159.7, 144.1, 134.1, 115.5, 115.2, 111.7, 105.1, 96.1, 68.3, 55.2, 37.5, 23.9, 9.8, 0.0. HRMS (ESI): m/z [M + H]$^+$ calcd for $C_{17}H_{25}O_2Si^+$: 289.1618; found: 289.1624.

Synthesis of 7

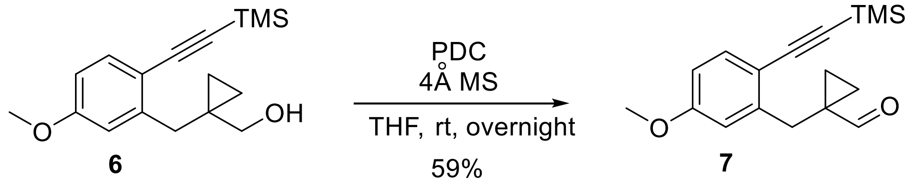

To a solution of **6** (4.10 g, 14.23 mmol, 1.0 equiv) in 140 ml DCM was added PDC (8.46 g, 28.46 mmol, 2.0 equiv) and 4Å MS (8.46 g, the mass as same as the PDC) at rt. Then the reaction mixture was stirred overnight. After filtering the solid, the resulting solution was concentrated by rotary evaporation. Purification of the crude product by flash column chromatography (silica gel, 15:1–10:1 petroleum ether/EtOAc) afforded the target compound **7** (2.43 g, 59%) as pale-yellow oil.

TLC (10:1 PE/EA, R_f): 0.4

^{1}H NMR (400 MHz, $CDCl_3$) δ = 8.75 (s, 1H), 7.36 (d, J = 8.6 Hz, 1H), 6.87 (d, J = 2.6 Hz, 1H), 6.69 (dd, J = 8.5, 2.7 Hz, 1H), 3.79 (s, 3H), 3.23 (s, 2H), 1.16 – 1.11 (m, 4H), 0.22 (s, 9H). **^{13}C NMR (101 MHz, $CDCl_3$)** δ = 201.4, 159.6, 143.2, 133.6, 115.4, 115.4, 111.9, 104.2, 96.5, 55.2, 32.7, 32.6, 12.9, 0.0.

HRMS (ESI): m/z $[M + H]^+$ calcd for $C_{17}H_{23}O_2Si^+$: 287.1462; found: 287.1469.

Synthesis of 8

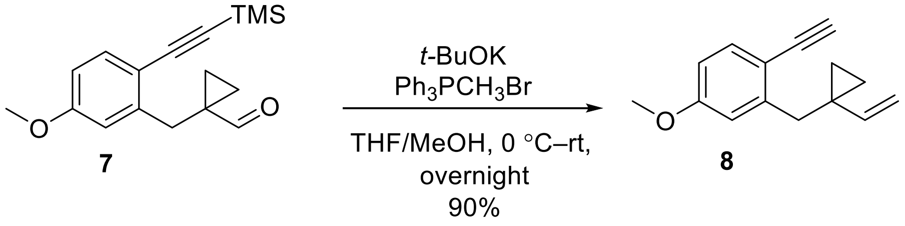

To a solution of *t*-BuOK (1.33 g, 11.88 mmol, 2.0equiv) and Ph_3PCH_3Br (4.24 g, 11.88 mmol, 2.0 equiv) in THF (60 mL) was added **7** (1.70 g, 5.94 mmol, 1.0 equiv) in THF (15 mL) under argon at 0 °C and the resulting reaction mixture was stirred at rt for 6 h. After that, MeOH (30 mL) was added under air and the reaction mixture was stirred overnight, then quenched with saturated NH_4Cl, extracted with EA. The combined

organic layers were washed with brine, dried over anhydrous Na_2SO_4, filtered, and concentrated by rotary evaporation. Purification of the crude product by flash column chromatography (silica gel, 100:1–80:1 petroleum ether/EtOAc) afforded the target compound **8** (1.13 g, 90%) as a pale-yellow oil.

TLC (PE, R_f): 0.3

^{1}H NMR (400 MHz, $CDCl_3$) δ = 7.41 (d, J = 8.5 Hz, 1H), 6.95 (d, J = 2.7 Hz, 1H), 6.69 (dd, J = 8.5, 2.7 Hz, 1H), 5.65 (dd, J = 17.2, 10.7 Hz, 1H), 4.91 – 4.85 (m, 2H), 3.79 (s, 3H), 3.20 (s, 1H), 3.03 (s, 2H), 0.73 (s, 4H). **^{13}C NMR (101 MHz, $CDCl_3$)** δ = 159.6, 144.1, 143.8, 133.9, 115.2, 114.4, 111.2, 111.0, 82.6, 79.9, 55.2, 38.6, 21.5, 13.8. HRMS (ACPI): m/z $[M + H]^+$ calcd for $C_{15}H_{17}O^+$: 213.1274; found: 213.1272.

Synthesis of 9

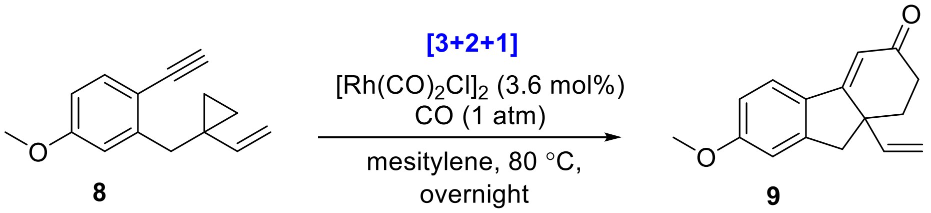

To a solution of **8** (4.37 g, 20.6 mmol, 1.0 equiv) in mesitylene (206 mL) was added $[Rh(CO)_2Cl_2]$ (287.8 mg, 0.74 mmol, 0.036 equiv). The reaction mixture was charged with CO three times and bubbled with balloon-pressured (slightly higher than 1 atm) gas of CO at room temperature for 5 min and then stirred at 80 °C overnight under balloon-pressured gas of CO. Then removing solvent by rotary evaporation and purification of the crude product by flash column chromatography (silica gel, 10:1–8:1 petroleum ether/EtOAc) afforded the target compound **9** (4.29 g, 87%) as an orange solid.

TLC (5:1 PE/EA, R_f): 0.4

^{1}H NMR (400 MHz, $CDCl_3$) δ = 7.51 (d, *J* = 8.5 Hz, 1H), 6.83 (dd, *J* = 8.5, 2.4 Hz, 1H), 6.80 (d, *J* = 2.4 Hz, 1H), 6.27 (s, 1H), 5.86 (dd, *J* = 17.2, 10.4 Hz, 1H), 5.02 (dd,

J = 10.4, 0.9 Hz, 1H), 4.93 (dd, J = 17.3, 1.0 Hz, 1H), 3.81 (s, 3H), 3.03 – 2.91 (m, 2H), 2.54 – 2.36 (m, 2H), 2.25 – 2.07 (m, 2H). **^{13}C NMR (101 MHz, CDCl$_3$)** δ = 199.3, 167.5, 163.1, 148.4, 140.8, 130.7, 124.1, 116.9, 115.3, 114.5, 110.0, 55.4, 50.9, 45.1, 34.4, 33.3.

M P: 92.9-94.9 °C.

HRMS (ESI): m/z [M + H]$^+$ calcd for $C_{16}H_{17}O_2^+$: 241.1223; found: 241.1228.

Synthesis of 10[2]

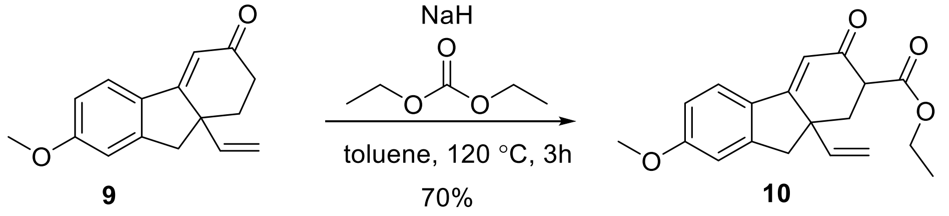

To a solution of **9** (120.0 mg, 0.5 mmol, 1.0 equiv) in dry toluene (5 mL) was added diethyl carbonate (0.18 mL, 1.5 mmol, 3.0 equiv) and NaH (60 mg, 60% ,1.5 mmol, 3.0 equiv) under argon. The reaction mixture was stirred at 120 °C for 3 h. After cooling, the mixture was neutralised by 1 N hydrochloric acid, then poured into brine, extracted by EA. The combined organic layer was dried over anhydrous Na_2SO_4, filtered, and concentrated by rotary evaporation. Purification of the crude product by flash column chromatography (silica gel, 10:1 petroleum ether/EtOAc) afforded the target compound **10** (109.3 mg, 70%) as a yellow solid. (dr = 4.5:1)

TLC (5:1 PE/EA, R$_f$): 0.5

^{1}H NMR (400 MHz, CDCl$_3$) Major: δ = 7.52 (d, J = 8.6 Hz, 1H), 6.89 – 6.79 (m, 2H), 6.31 (s, 1H), 5.89 (dd, J = 17.2, 10.4 Hz, 1H), 5.05 (dd, J = 10.3, 0.8 Hz, 1H), 4.93 (dd, J = 17.3, 0.9 Hz, 1H), 4.29-4.17 (m, 2H), 3.83 (d, J = 2.2 Hz, 3H), 3.51 (dd, J = 13.1, 4.7 Hz, 1H), 3.10 – 2.98 (m, 2H), 2.52 (t, J = 12.9 Hz, 1H), 2.46 – 2.39 (m, 1H), 1.31 (dt, J = 14.3, 7.2 Hz, 3H). Minor: δ= 7.52 (d, J = 8.6 Hz, 1H), 6.89 – 6.79 (m, 2H), 6.31 (s, 1H), 5.60 (dd, J = 17.2, 10.4 Hz, 1H), , 4.90 – 4.82 (m, 2H), 4.29-4.17 (m, 2H), 3.83

(overlap, 4H), 3.10 – 2.98 (m, 2H), 2.77 (d, J = 15.9 Hz, 1H), 2.39 (d, J = 2.3 Hz, 1H), 1.31 (dt, J = 14.3, 7.2 Hz, 3H). **^{13}C NMR (101 MHz, CDCl$_3$)** δ = 193.7, 170.9, 167.6, 163.4, 161.9, 156.8, 149.1, 148.2, 140.6, 140.3, 130.5, 130.4, 124.3, 123.6, 116.2, 115.8, 114.8, 114.4, 112.8, 110.0, 109.9, 109.8, 92.2, 61.2, 60.1, 55.5, 55.4, 51.3, 50.5, 47.6, 45.1, 45.0, 36.6, 31.6, 14.4, 14.1.

M P: 84.3-86.8 °C.

HRMS (ESI): m/z [M + H]$^+$ calcd for $C_{19}H_{21}O_4^+$: 313.1434; found: 313.1443.

Synthesis of 11[3]

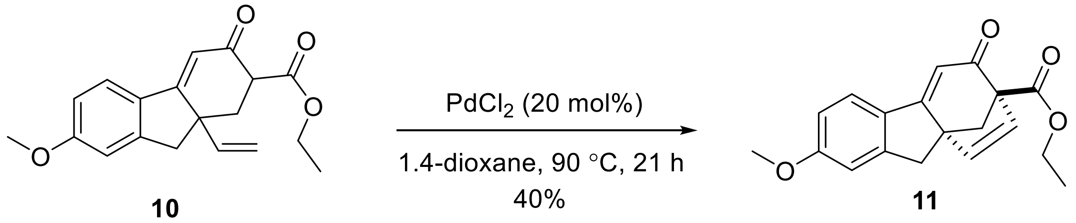

To a flask were added **10** (93.6 mg, 0.3 mmol, 1.0 equiv), $PdCl_2$ (10.6 mg, 0.06 mmol, 0.2 equiv) and dioxane (9 mL). The reaction mixture was stirred at 90 °C overnight. After that, the reaction mixture was cooled to room temperature and concentrated. The crude mixture was purified by flash column chromatography with silica gel (eluted with petroleum ether/ EtOAc = 15:1–10:1) to afford the pure product **11** (37.0 mg, 40%) as a yellow solid.

TLC (5:1 PE/EA, R$_f$): 0.4

^{1}H NMR (400 MHz, CDCl$_3$) δ = 7.49 (d, J = 9.3 Hz, 1H), 6.90 (d, J = 7.7 Hz, 2H), 6.43 (d, J = 5.2 Hz, 1H), 6.34 (d, J = 5.3 Hz, 1H), 5.74 (s, 1H), 4.29 (q, J = 7.1 Hz, 2H), 3.87 (s, 3H), 3.26 (d, J = 17.6 Hz, 1H), 3.08 (d, J = 17.6 Hz, 1H), 2.87 – 2.77 (m, 2H), 1.32 (t, J = 7.1 Hz, 3H). **^{13}C NMR (101 MHz, CDCl$_3$)** δ = 193.4, 171.6, 170.1, 163.6, 151.1, 146.0, 133.3, 128.3, 124.7, 115.5, 109.7, 107.2, 70.7, 61.2, 57.3, 56.5, 55.6, 39.0, 14.2.

M P: 119.9-121.6 °C.

HRMS (ESI): m/z $[M + H]^+$ calcd for $C_{19}H_{19}O_4^+$: 311.1278; found: 311.1286.

Note: We have also performed the Heck cyclization experiments as follows: Under standard conditions, using 1 equivalent of $PdCl_2$, the product was obtained in a yield of 37%. The scale of this reaction was 0.143 mmol, and the mass of the product was 16.5 mg. In another experiment, under an O_2 atmosphere and standard conditions, the product was obtained in 24%. The scale of this reaction was 0.142 mmol, and the mass of the product was 10.4 mg. We did not know the reason for this and did not investigate this further.

Synthesis of 12

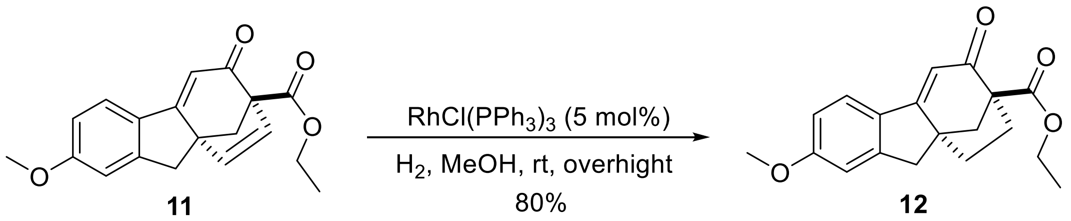

To a flask were added **11** (119.3 mg, 0.385 mmol, 1.0 equiv), $Rh(PPh_3)_3Cl$ (17.9 mg, 0.0193 mmol, 0.05 equiv) and MeOH (6.2 mL), Then the reaction mixture was charged with H_2 three times. The reaction mixture was then stirred at rt overnight. The reaction mixture was then concentrated by rotary evaporation. The crude mixture was purified by flash column chromatography with silica gel (eluted with petroleum ether/ EtOAc = 15:1–10:1) to afford the pure product **12** (96.3 mg, 80%) as a pale-yellow solid.

TLC (3:1 PE/EA, R*f*): 0.5

^{1}H NMR (400 MHz, CDCl$_3$) δ = 7.50 (d, *J* = 9.3 Hz, 1H), 6.88 (d, *J* = 7.5 Hz, 2H), 6.09 (s, 1H), 430-3.86 (m, 2H), 3.86 (s, 3H), 3.23 (d, *J* = 17.1 Hz, 1H), 3.03 (d, *J* = 17.0 Hz, 1H), 2.68 – 2.57 (m, 1H), 2.35 (d, *J* = 11.1 Hz, 1H), 2.20 (d, *J* = 11.2 Hz, 1H), 2.05 – 1.87 (m, 3H), 1.30 (t, *J* = 7.1 Hz, 3H). **^{13}C NMR (101 MHz, CDCl$_3$)** δ = 198.4, 173.5, 172.1, 163.4, 150.8, 128.5, 125.0, 115.0, 111.3, 109.8, 64.6, 60.9, 55.5, 52.9, 45.9, 40.0, 36.5, 28.7, 14.1.

M P: 90.1-92.5 °C.

HRMS (ESI): m/z $[M + H]^+$ calcd for $C_{19}H_{21}O_4^+$: 313.1434; found: 313.1442.

Synthesis of 13

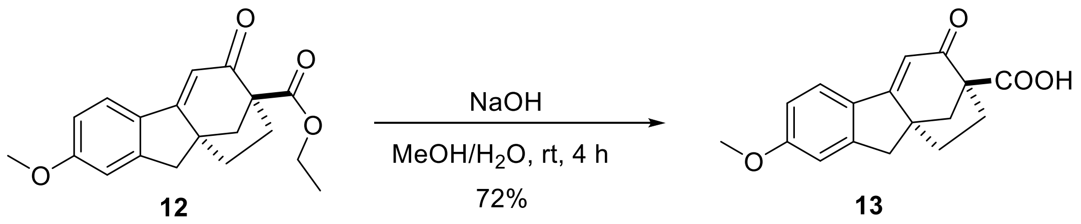

To a solution of **13** (206.4 mg, 0.66 mmol, 1.0 equiv) in MeOH (20 mL) was slowly added the cooled NaOH (1.32 g, 33 mmol, 50 equiv) in H_2O (6 mL) and stirred at rt for 4 h. After completion of the reaction, the pH was adjusted to acidic by acidification using 1 N hydrochloric acid. Extract with ethyl acetate three times, and then the organic layer was dried by Na_2SO_4, the solution was concentrated by rotary evaporation. Then the crude mixture was purified by flash column chromatography with silica gel (eluted with EtOAc/petroleum ether = 1:1–2:1) to afford the product **13** (134.7 mg, 72%) as a pale-yellow solid.

TLC (1:3 PE/EA, R_f): 0.1

^{1}H NMR (400 MHz, $CDCl_3$) δ = 7.54 (d, J = 8.6 Hz, 1H), 6.91 (d, J = 7.7 Hz, 2H), 6.21 (s, 1H), 3.87 (s, 3H), 3.33 – 3.10 (m, 2H), 2.68 (d, J = 11.4 Hz, 1H), 2.63-2.56 (m, 1H), 2.24-2.17 (m, 1H), 2.08 (d, J = 11.1 Hz, 1H), 2.03-1.96 (m, 1H), 1.87 (d, J = 11.3 Hz, 1H). **^{13}C NMR (101 MHz, $CDCl_3$)** δ = 203.4, 178.7, 174.4, 164.5, 152.4, 127.8, 125.7, 115.6, 110.7, 109.8, 58.0, 55.6, 54.2, 46.5, 39.8, 36.4, 33.9.

M P: 187.8-188.9 °C.

HRMS (ESI): m/z $[M + H]^+$ calcd for $C_{17}H_{17}O_4^+$: 285.1121; found: 285.1129.

Synthesis of 1[4]

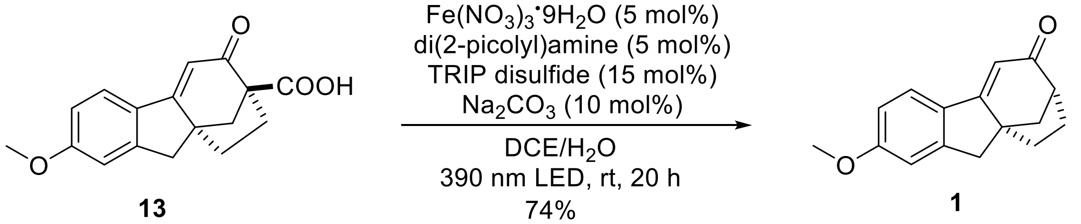

13 **1**

Acid **15** (28.4 mg, 0.1 mmol, 1.0 equiv), $Fe(NO_3)_3$•$9H_2O$ (2 mg, 0.005 mmol, 0.05 equiv), di(2-picolyl)amine (1 mg, 0.005 mmol, 0.05 equiv), Na_2CO_3 (1 mg, 0.01 mmol, 0.1 equiv) and TRIP disulfide (7 mg, 0.015 mmol, 0.15 equiv) were added to a flask. Then DCE (0.5 mL) and H_2O (0.5 mL) were added to the flask in the glovebox. The reaction flask then moved outside the glove box and stirred vigorously for an extra 5 min before shining the light to make sure all solids have dissolved. The sample was then irradiated with a 390 nm LED Kessil lamp (5 to 10 cm to the reaction vial) and stirred for 20 h at room temperature. After completion, the crude mixture was purified by flash column chromatography with silica gel (eluted with petroleum ether/ EtOAc = 10:1–3:1) to afford the pure product **1** (17.8 mg, 74%) as a pale yellow solid. All data of 1 agreed with the literature.[5]

^{1}H NMR (400 MHz, $CDCl_3$) δ = 7.49 (d, *J* = 9.2 Hz, 1H), 6.91 – 6.83 (m, 2H), 6.05 (s, 1H), 3.85 (s, 3H), 3.22 (d, *J* = 17.0 Hz, 1H), 3.04 (d, *J* = 17.4 Hz, 1H), 3.02 – 2.97 (m, 1H), 2.32-2.23 (m, 1H), 2.02 – 1.85 (m, 4H), 1.73-1.66 (m, 1H). **^{13}C NMR (101 MHz, $CDCl_3$)** δ = 204.0, 174.0, 163.2, 151.1, 128.9, 124.8, 114.8, 112.4, 109.8, 55.5, 53.1, 51.1, 43.5, 40.1, 36.8, 26.3.

M P: 110.5-112.6 °C.

HRMS (ESI): m/z $[M + H]^+$ calcd for $C_{16}H_{17}O^+$: 241.1223; found: 241.1228.

3. References

1. Ruiz, J.; Ardeo, A.; Ignacio, R.; Sotomayor, N.; Lete, E. An Efficient Entry to Pyrrolo[1,2-b]isoquinolines and Related Systems Through Parham Cyclisation. *Tetrahedron* .**2005**, *61*, 3311-3324.
2. Cui, L. Q.; Dong, Z. L.; Liu, K.; Zhang, C. Design, Synthesis, Structure, and Dehydrogenation Reactivity of a Water-Soluble o-Iodoxybenzoic Acid Derivative Bearing a Trimethylammonium Group. *Org. Lett.* **2011**, *13,* 6488-6491.
3. Han, X.; Wang, X.; Pei, T.; Widenhoefer, R. A. Palladium-Catalyzed Intramolecular Hydroalkylation of Alkenyl- b-Keto Esters, a-Aryl Ketones, and Alkyl Ketones in the Presence of Me_3SiCl or HCl. *Chemistry A European J.* **2004**, *10*, 6333-6342.
4. Lu, Y.; West, J. G. Chemoselective Decarboxylative Protonation Enabled by Cooperative Earth-Abundant Element Catalysis. *Angew Chem Int Ed.* **2022**, *62*, e202213055.
5. Wallace, D. J.; Reamer, R. A. New Synthesis of a Selective Estrogen Receptor Modulator Using an Enatioselective Phosphine-mediated 2+3 Cycloaddition. *Tetrahedron Lett.* .**2013**, *54*, 4425-4428.

4. NMR spectra of products 1–13

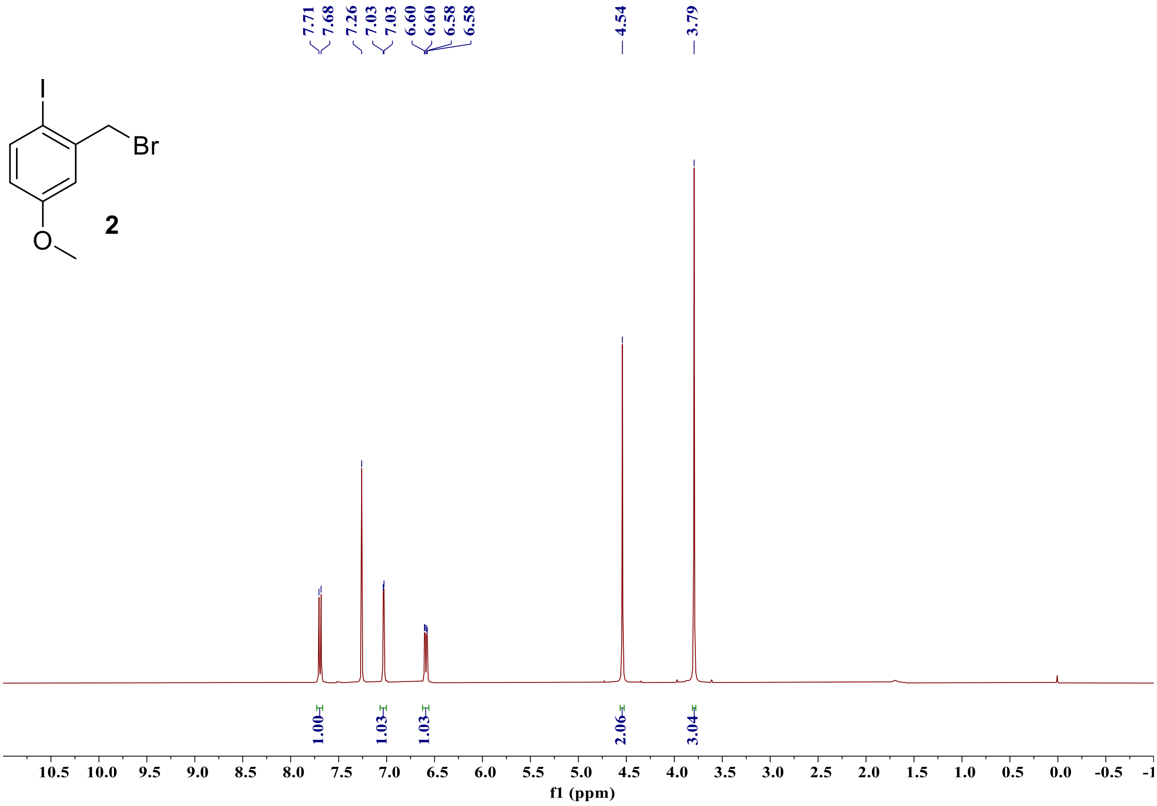

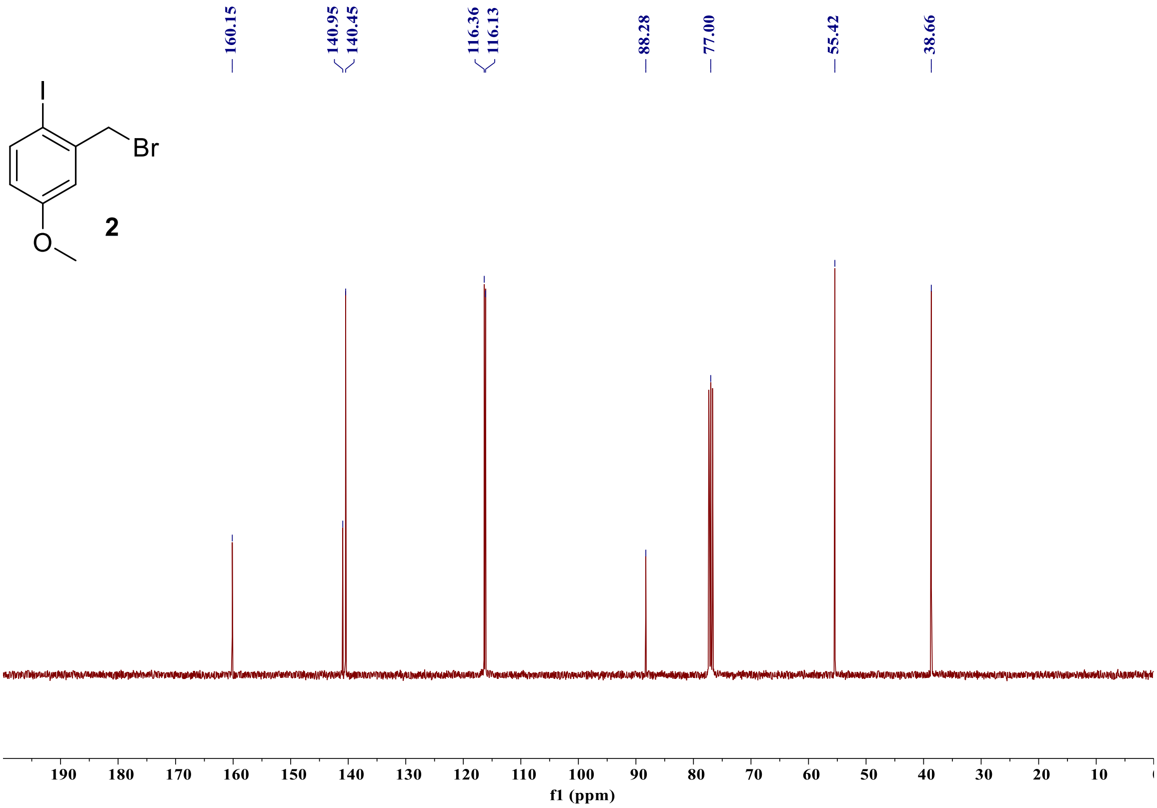

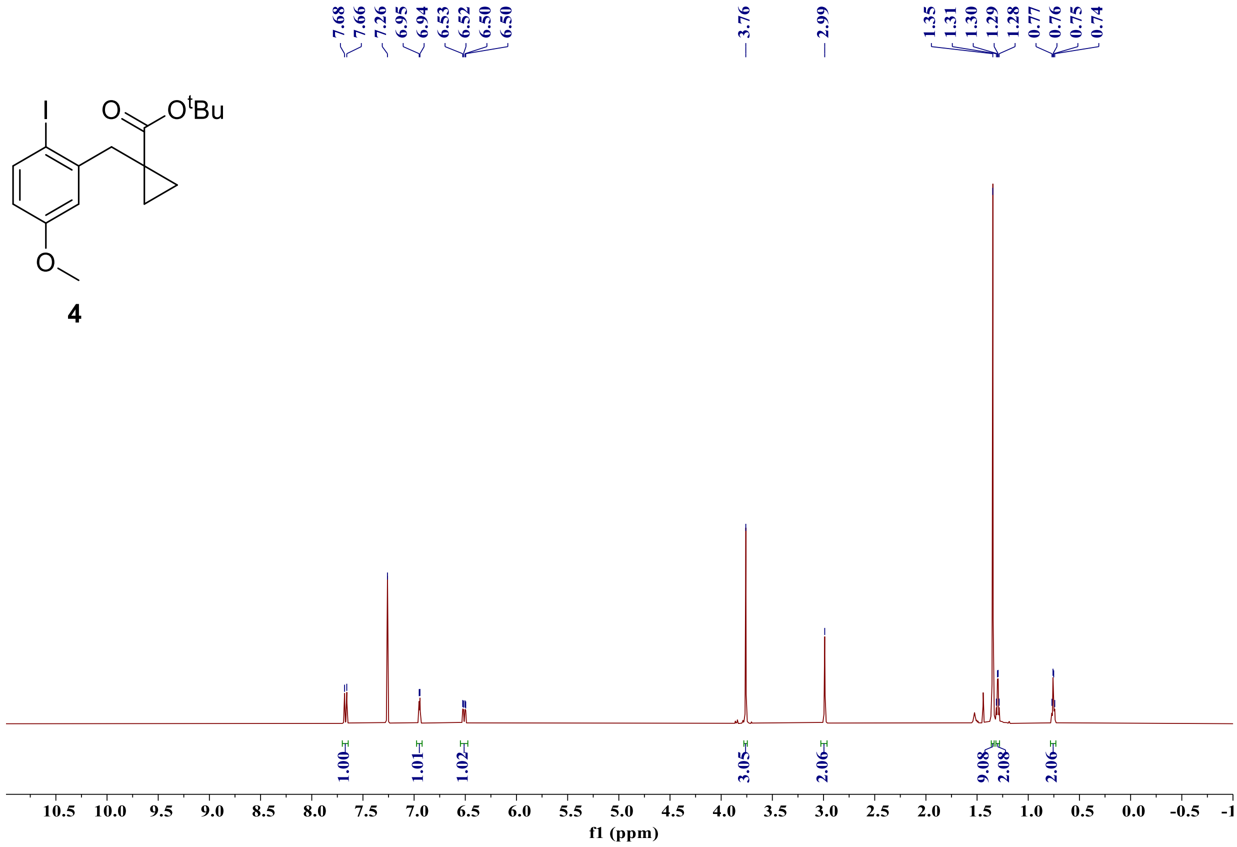
7.68
7.66
7.26
6.95
6.94
6.53
6.52
6.50
6.50
3.76
2.99
1.35
1.31
1.30
1.29
1.28
0.77
0.76
0.75
0.74
O
O^tBu
I
O
4
1.00
1.01
1.02
3.05
2.06
9.08
2.08
2.06
f1 (ppm)

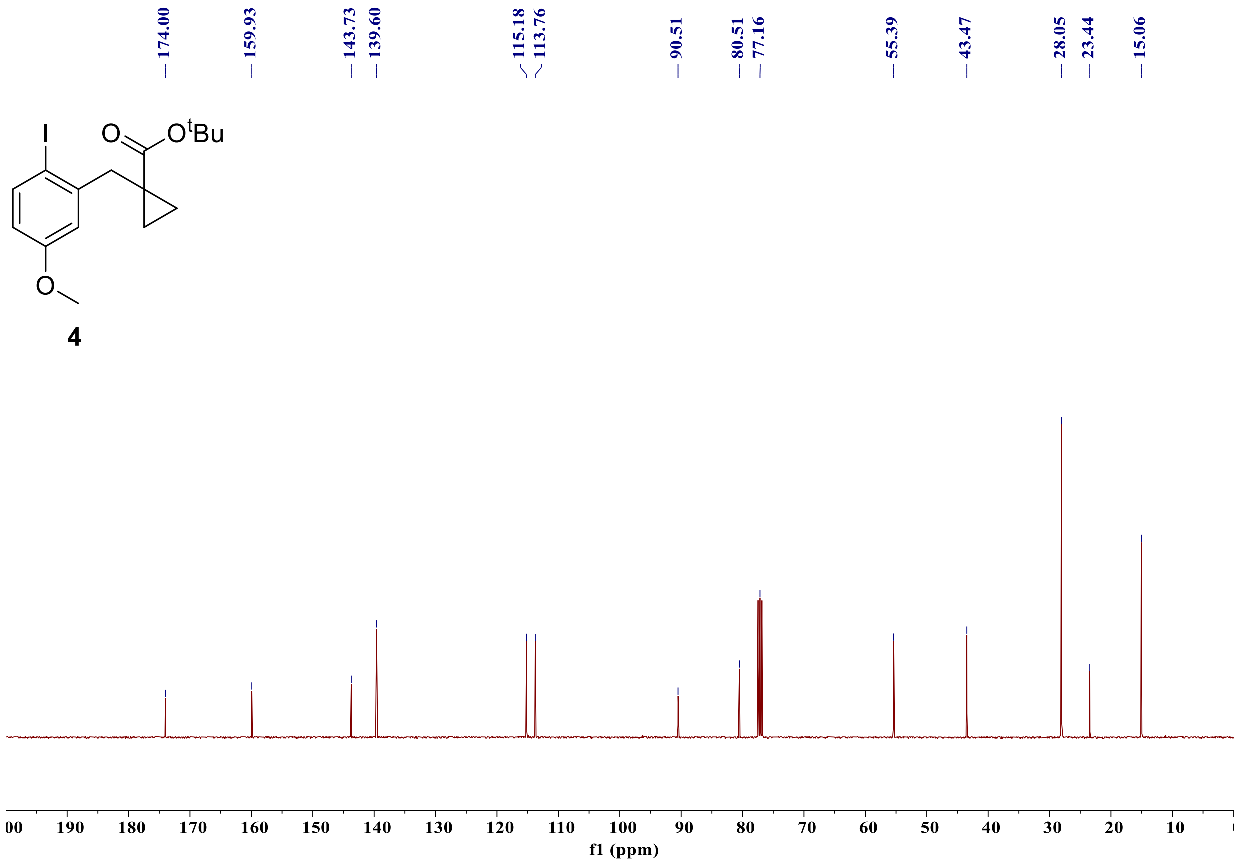
174.00
159.93
143.73
139.60
115.18
113.76
90.51
80.51
77.16
55.39
43.47
28.05
23.44
15.06
O
O^tBu
I
O
4
f1 (ppm)

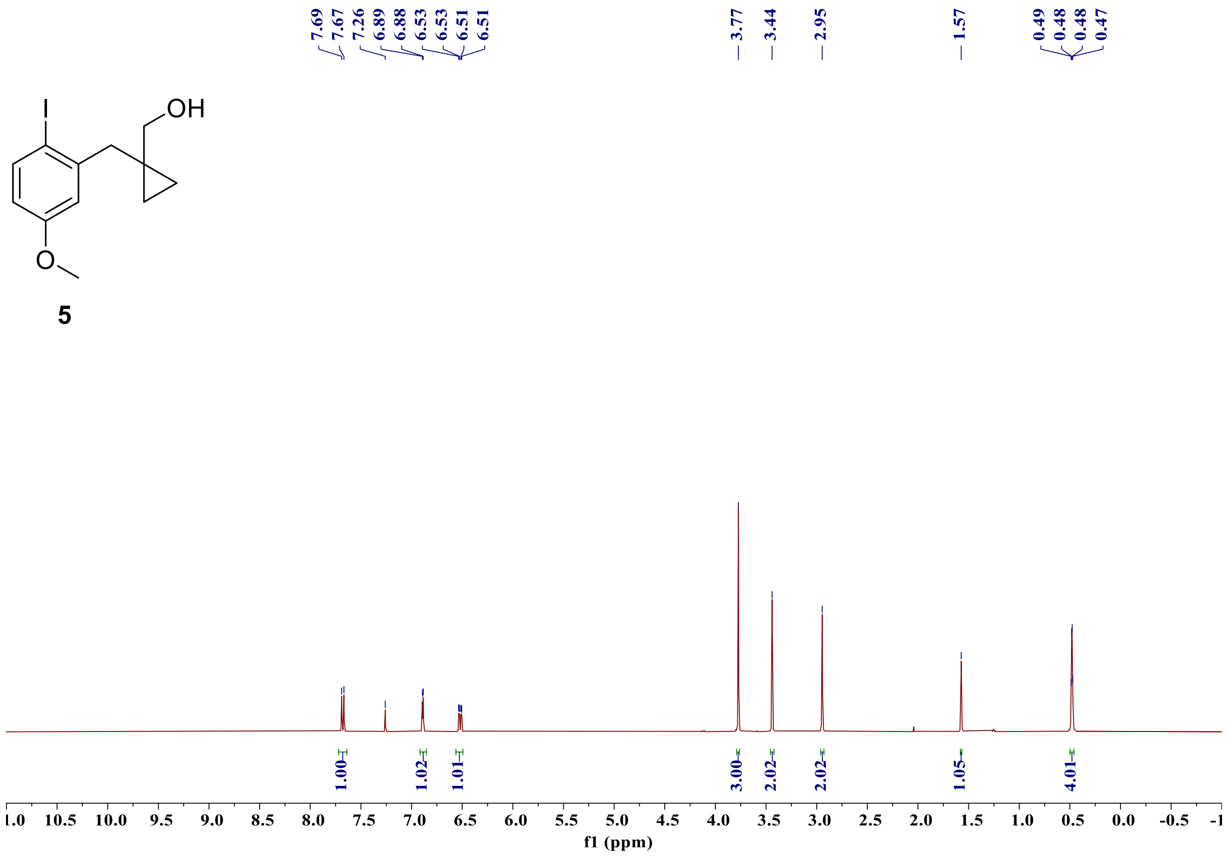
7.69
7.67
7.26
6.89
6.88
6.53
6.53
6.51
6.51
3.77
3.44
2.95
1.57
0.49
0.48
0.48
0.47
OH
I
O
5
1.00
1.02
1.01
3.00
2.02
2.02
1.05
4.01
f1 (ppm)

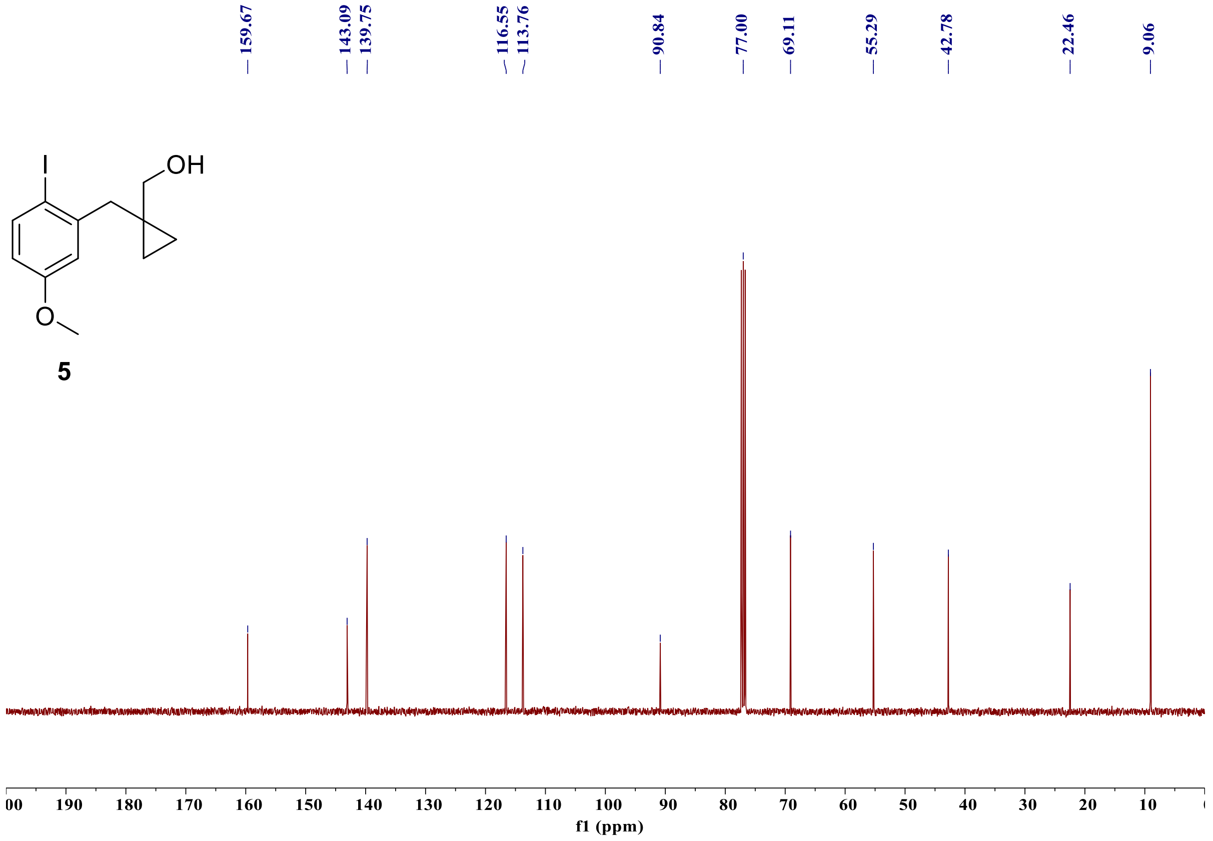
159.67
143.09
139.75
116.55
113.76
90.84
77.00
69.11
55.29
42.78
22.46
9.06
OH
I
O
5
f1 (ppm)

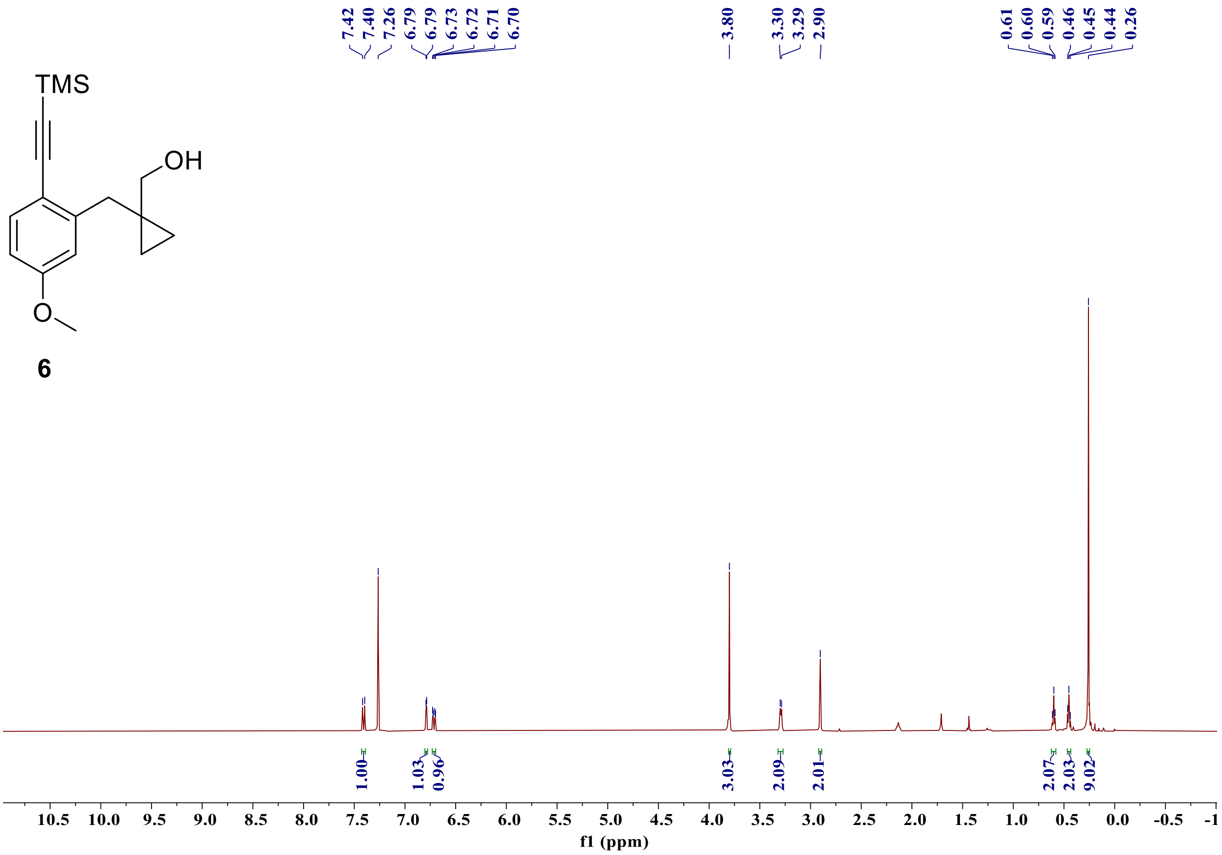
TMS
OH
O
6
7.42
7.40
7.26
6.79
6.79
6.73
6.72
6.71
6.70
3.80
3.30
3.29
2.90
0.61
0.60
0.59
0.46
0.45
0.44
0.26
1.00
1.03
0.96
3.03
2.09
2.01
2.07
2.03
9.02
f1 (ppm)

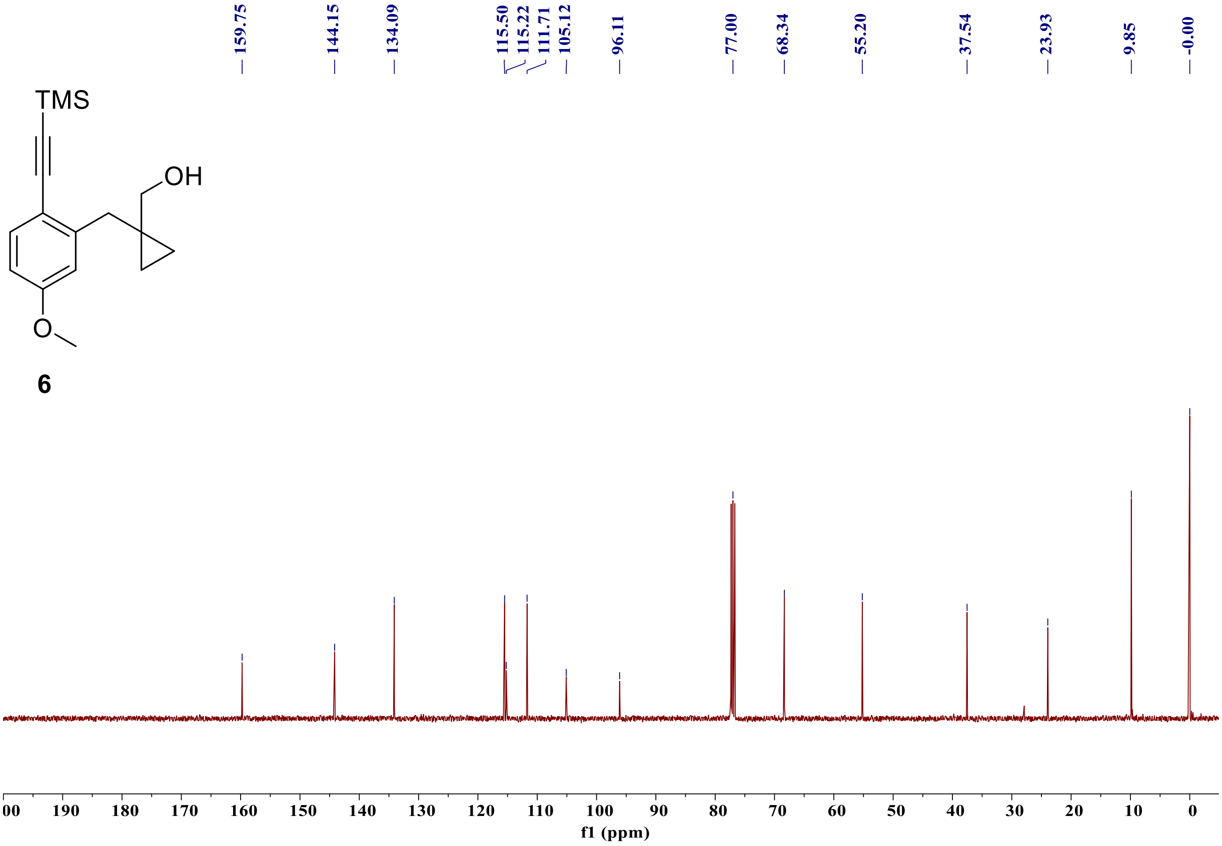
TMS
OH
O
6
159.75
144.15
134.09
115.50
115.22
111.71
105.12
96.11
77.00
68.34
55.20
37.54
23.93
9.85
-0.00
f1 (ppm)

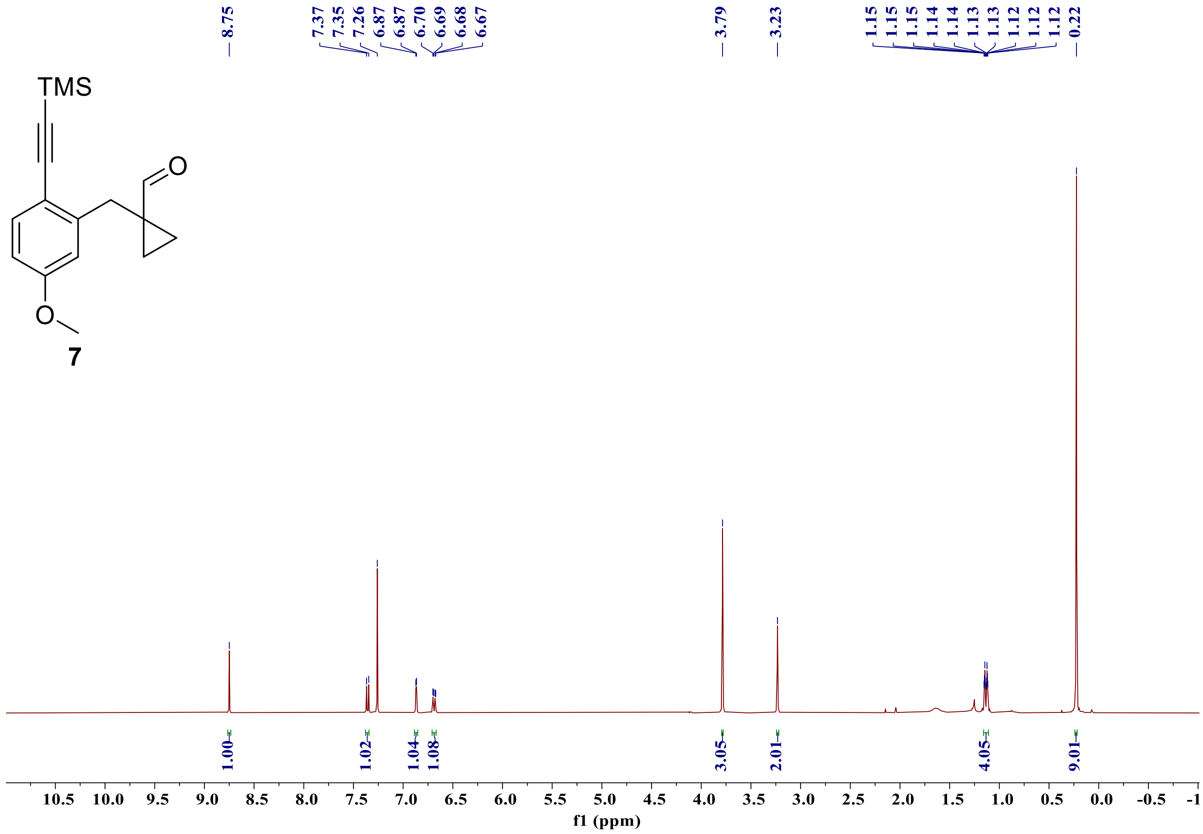
TMS
O
O
7
8.75
7.37
7.35
7.26
6.87
6.87
6.70
6.69
6.68
6.67
3.79
3.23
1.15
1.15
1.15
1.14
1.14
1.13
1.13
1.12
1.12
1.12
0.22
1.00
1.02
1.04
1.08
3.05
2.01
4.05
9.01
f1 (ppm)

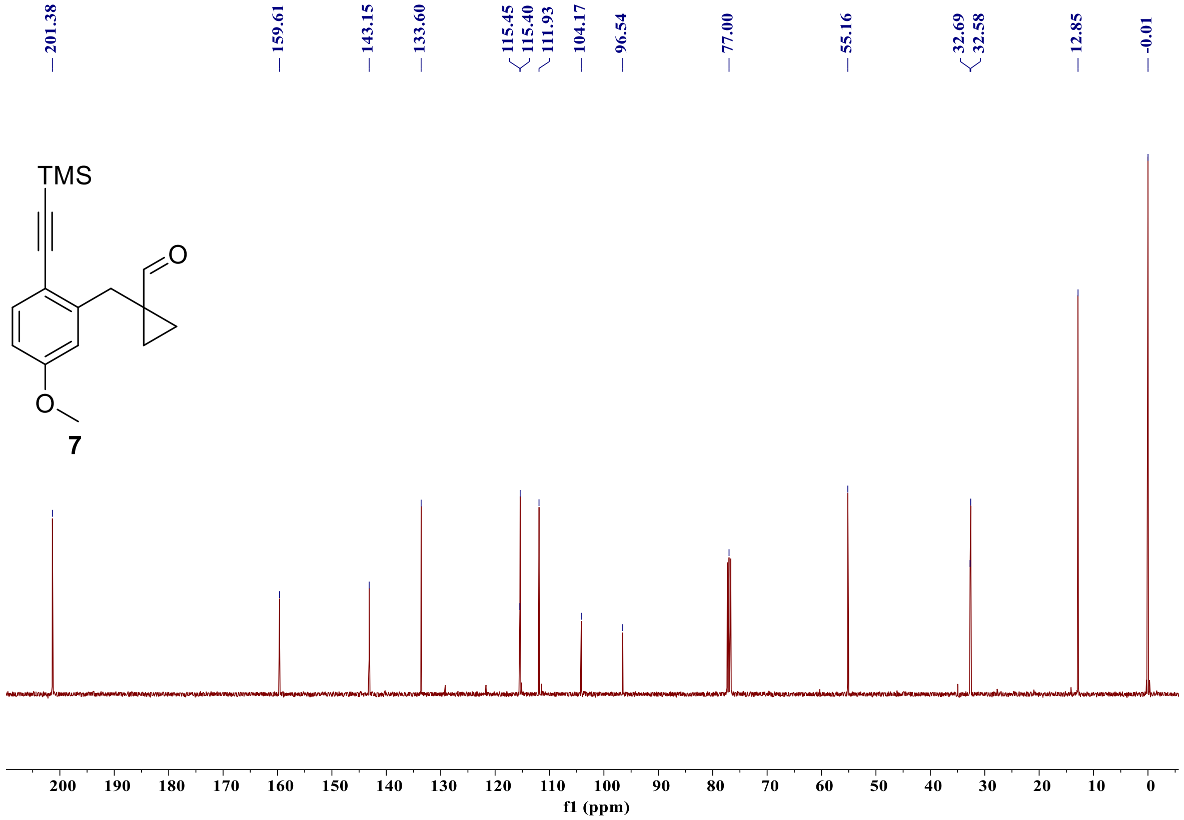
TMS
O
O
7
201.38
159.61
143.15
133.60
115.45
115.40
111.93
104.17
96.54
77.00
55.16
32.69
32.58
12.85
-0.01
f1 (ppm)

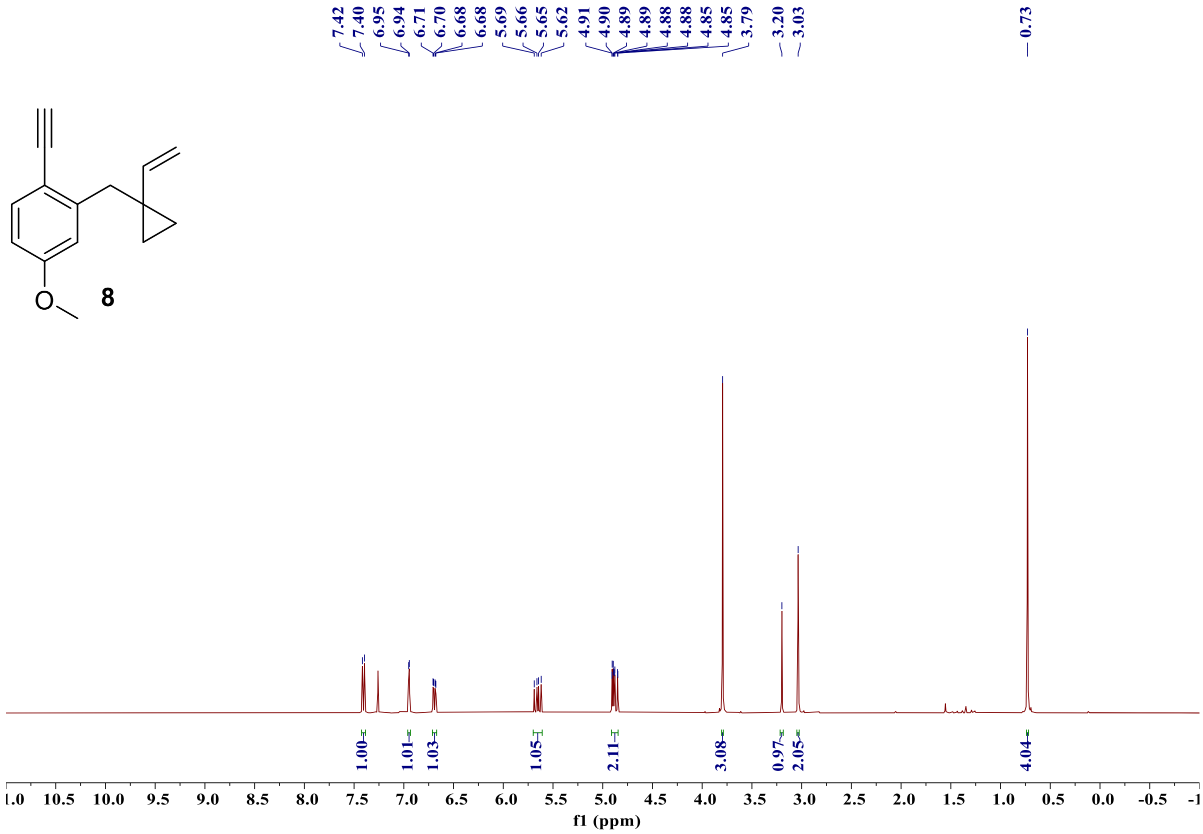
7.42
7.40
6.95
6.94
6.71
6.70
6.68
6.68
5.69
5.66
5.65
5.62
4.91
4.90
4.89
4.89
4.88
4.88
4.85
4.85
3.79
3.20
3.03
0.73
8
1.00
1.01
1.03
1.05
2.11
3.08
0.97
2.05
4.04
f1 (ppm)

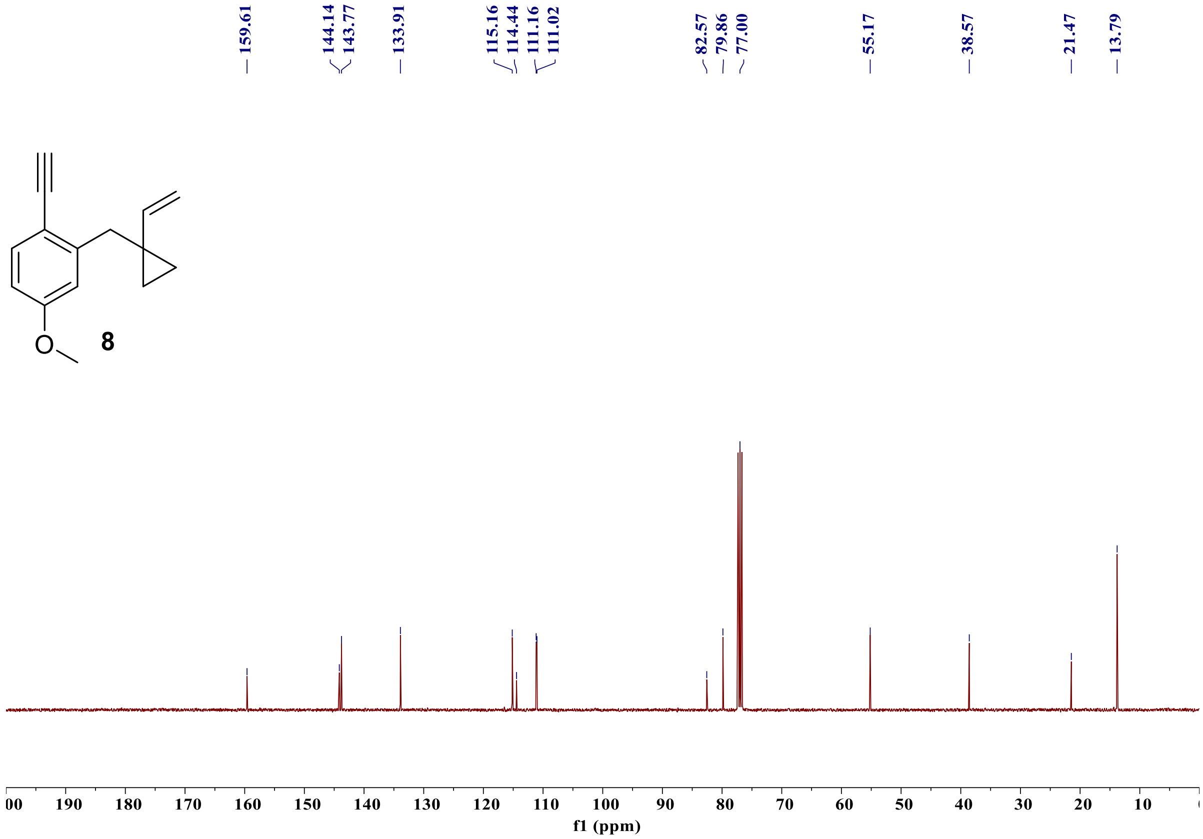
159.61
144.14
143.77
133.91
115.16
114.44
111.16
111.02
82.57
79.86
77.00
55.17
38.57
21.47
13.79
8
f1 (ppm)

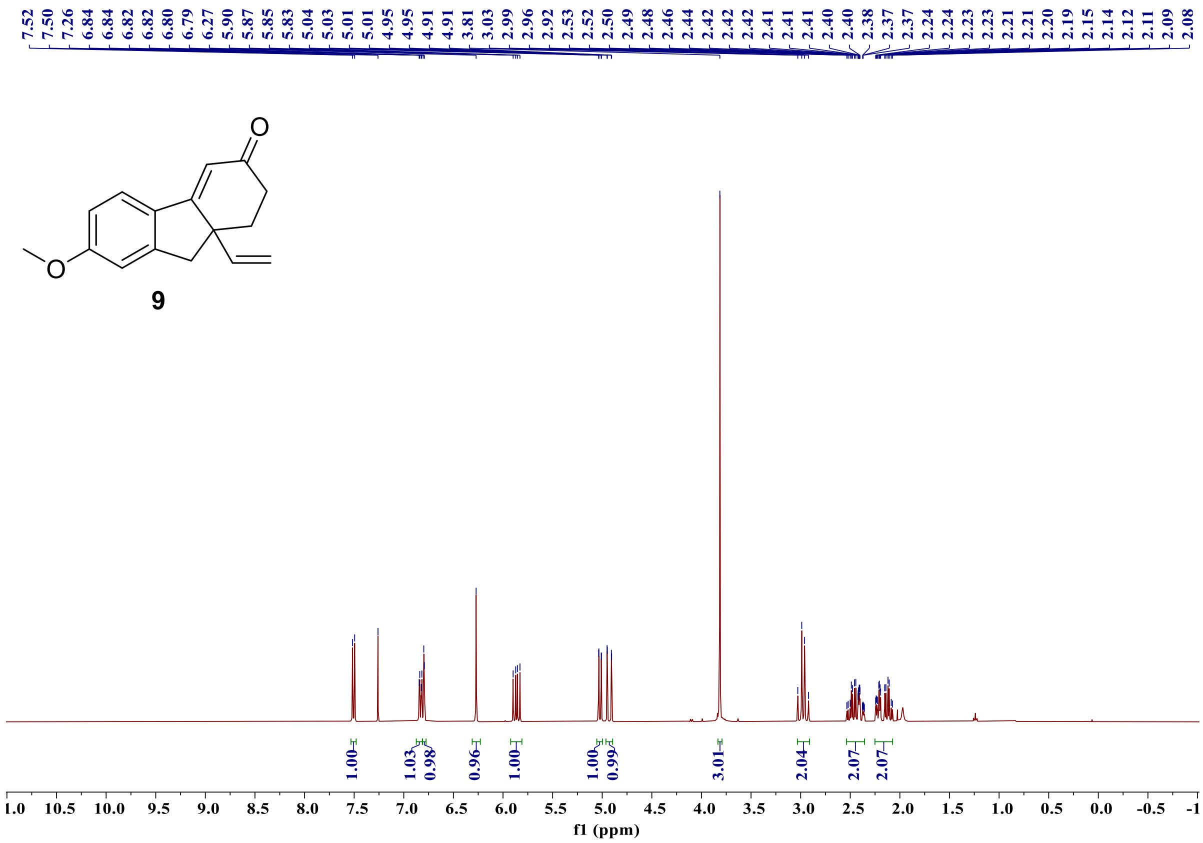

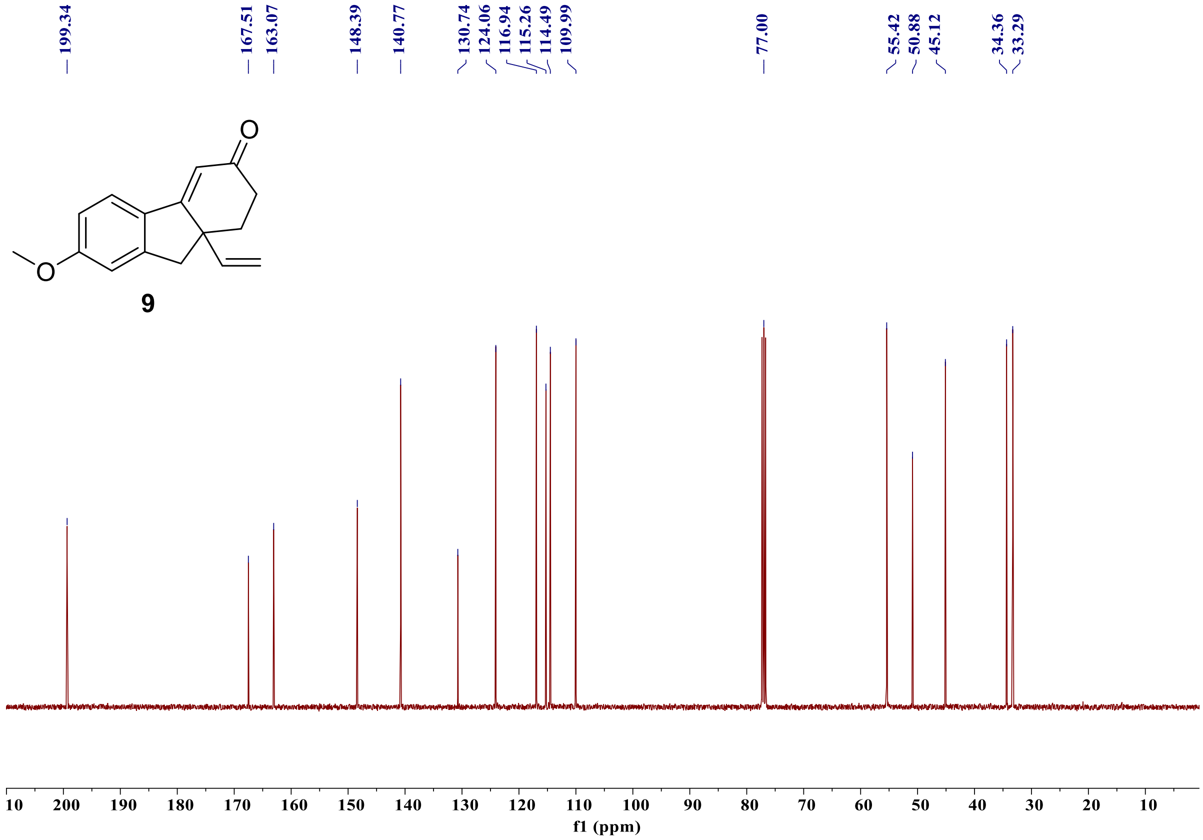

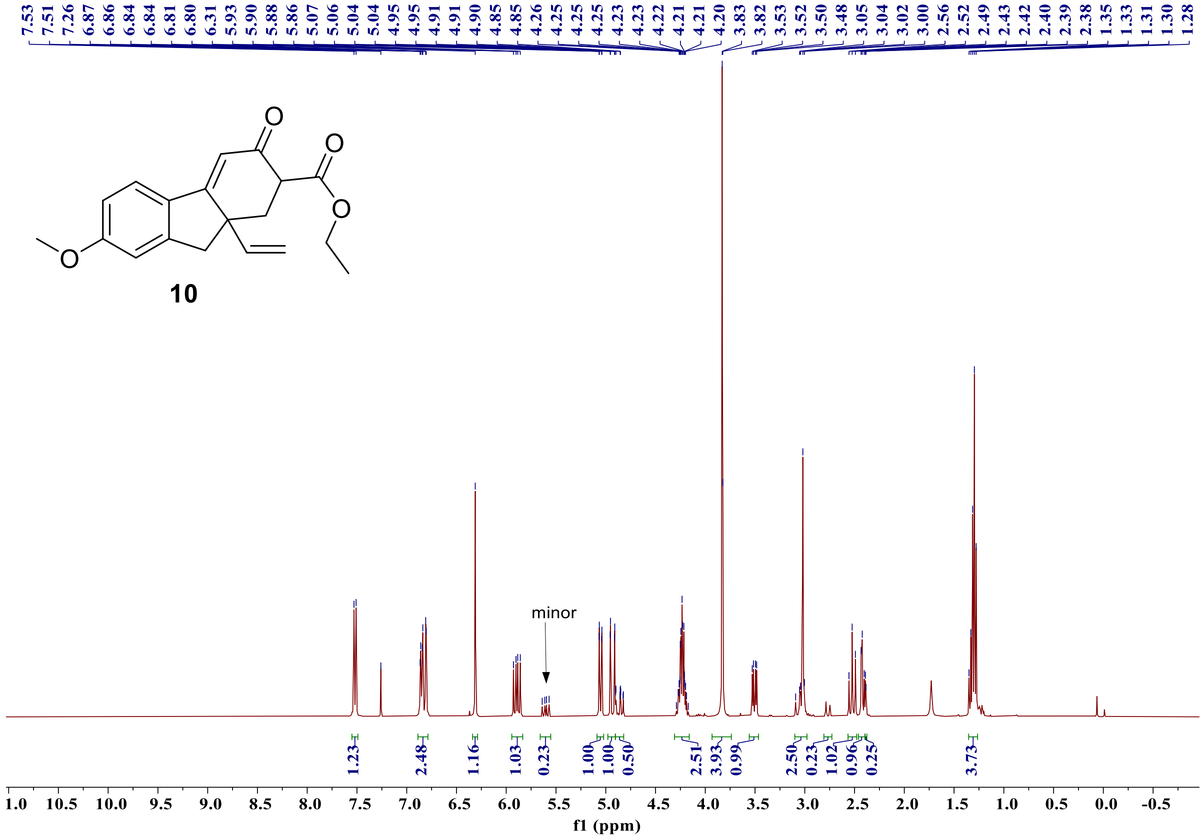
7.53
7.51
7.26
6.87
6.86
6.84
6.84
6.81
6.80
6.31
5.93
5.90
5.88
5.86
5.07
5.06
5.04
5.04
4.95
4.95
4.91
4.91
4.90
4.85
4.85
4.26
4.25
4.25
4.25
4.23
4.23
4.22
4.21
4.21
4.20
3.83
3.82
3.53
3.52
3.50
3.48
3.05
3.04
3.02
3.00
2.56
2.52
2.49
2.43
2.42
2.40
2.39
2.38
1.35
1.33
1.31
1.30
1.28
10
minor
1.23
2.48
1.16
1.03
0.23
1.00
1.00
0.50
2.51
3.93
0.99
2.50
0.23
1.02
0.96
0.25
3.73
1.0 10.5 10.0 9.5 9.0 8.5 8.0 7.5 7.0 6.5 6.0 5.5 5.0 4.5 4.0 3.5 3.0 2.5 2.0 1.5 1.0 0.5 0.0 -0.5
f1 (ppm)

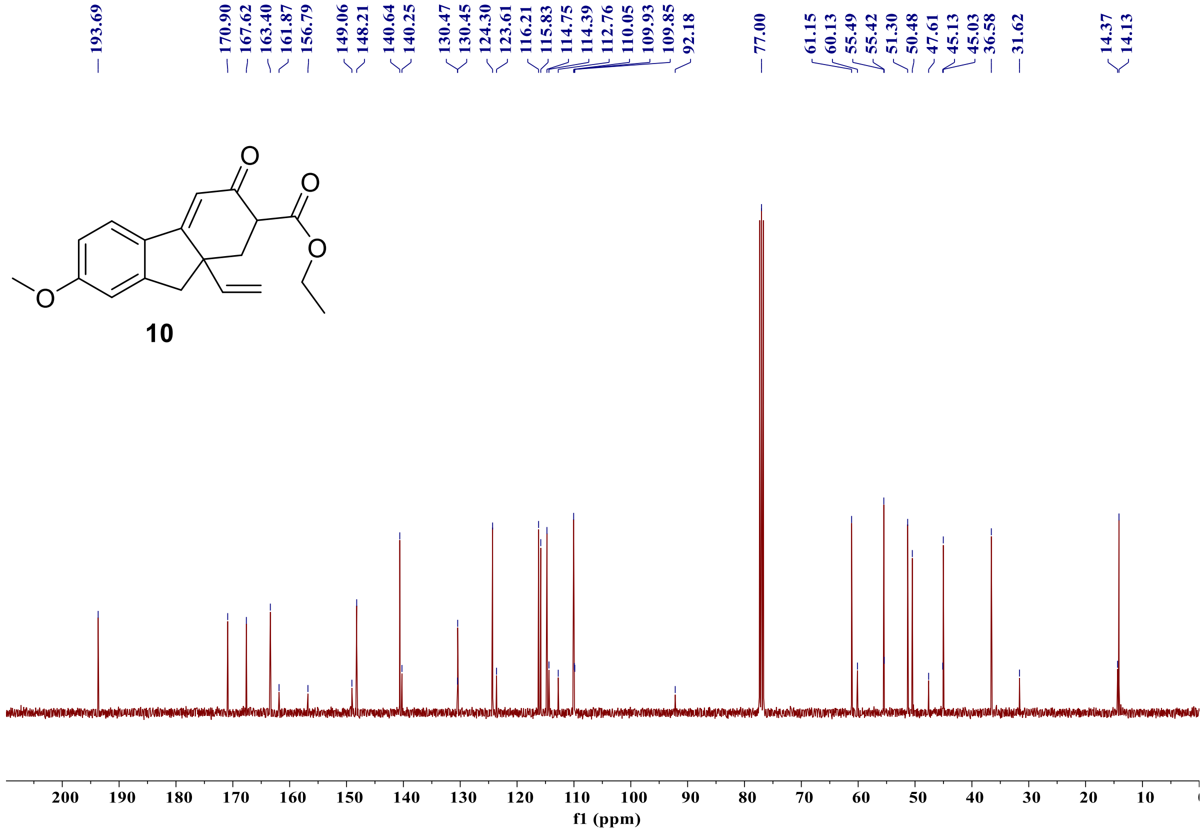
193.69
170.90
167.62
163.40
161.87
156.79
149.06
148.21
140.64
140.25
130.47
130.45
124.30
123.61
116.21
115.83
114.75
114.39
112.76
110.05
109.93
109.85
92.18
77.00
61.15
60.13
55.49
55.42
51.30
50.48
47.61
45.13
45.03
36.58
31.62
14.37
14.13
10
200 190 180 170 160 150 140 130 120 110 100 90 80 70 60 50 40 30 20 10
f1 (ppm)

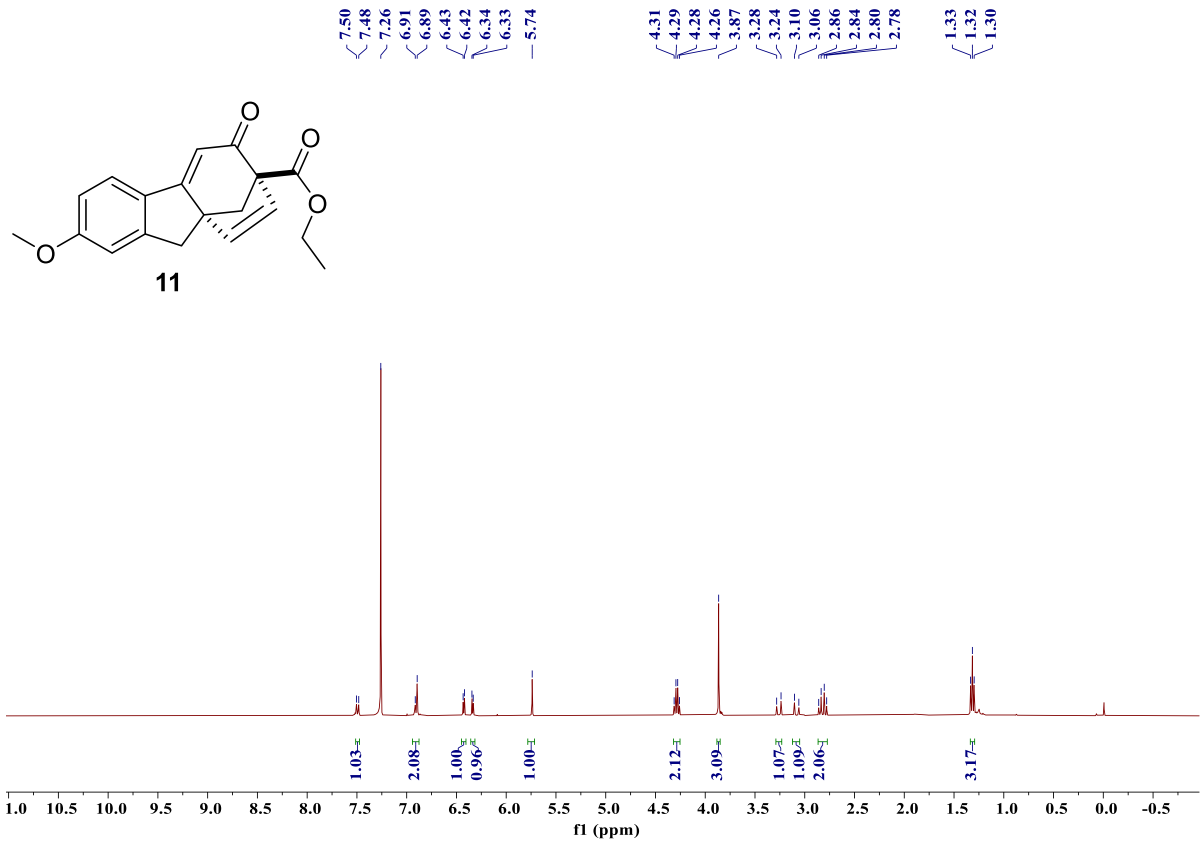
7.50
7.48
7.26
6.91
6.89
6.43
6.42
6.34
6.33
5.74
4.31
4.29
4.28
4.26
3.87
3.28
3.24
3.10
3.06
2.86
2.84
2.80
2.78
1.33
1.32
1.30
11
1.03
2.08
1.00
0.96
1.00
2.12
3.09
1.07
1.09
2.06
3.17
f1 (ppm)

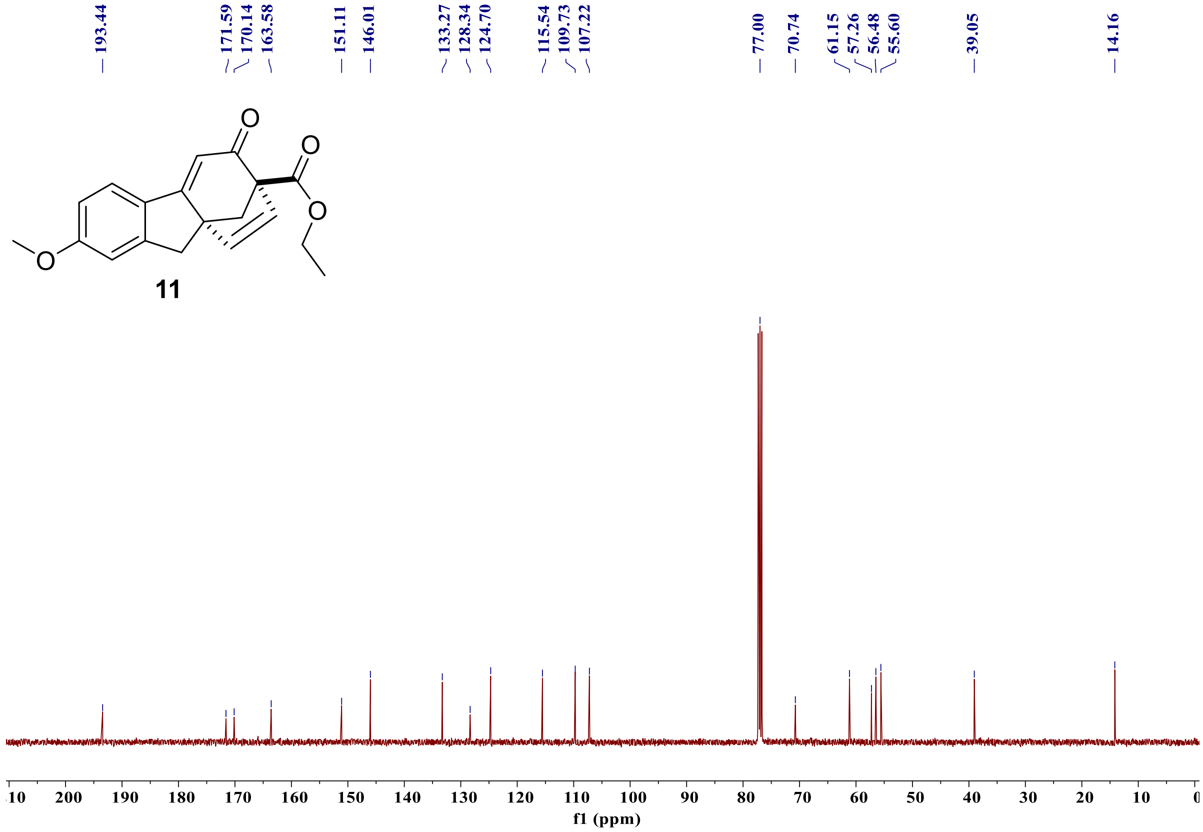
193.44
171.59
170.14
163.58
151.11
146.01
133.27
128.34
124.70
115.54
109.73
107.22
77.00
70.74
61.15
57.26
56.48
55.60
39.05
14.16
11
f1 (ppm)

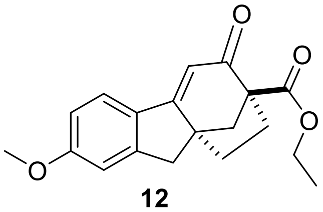

12

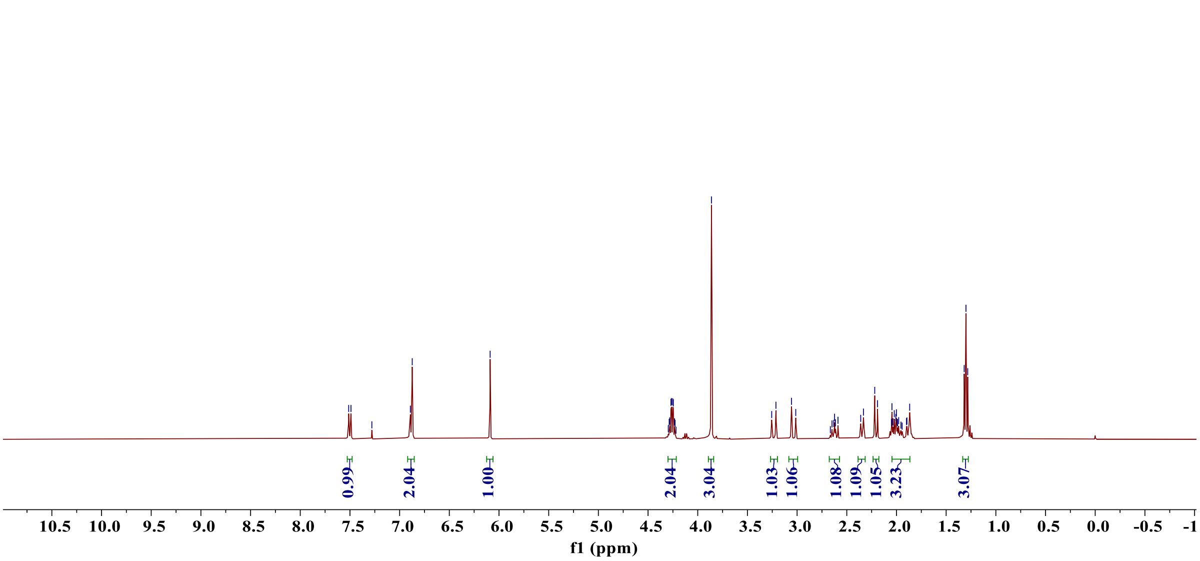
7.51
7.49
7.28
6.89
6.87
6.09
4.30
4.29
4.28
4.27
4.26
4.25
4.25
4.23
4.23
4.22
3.86
3.26
3.21
3.06
3.01
2.62
2.59
2.36
2.33
2.22
2.19
2.05
2.04
2.02
2.01
2.00
1.98
1.90
1.89
1.87
1.32
1.30
0.99
2.04
1.00
2.04
3.04
1.03
1.06
1.08
1.09
1.05
3.23
3.07
10.5 10.0 9.5 9.0 8.5 8.0 7.5 7.0 6.5 6.0 5.5 5.0 4.5 4.0 3.5 3.0 2.5 2.0 1.5 1.0 0.5 0.0 -0.5
f1 (ppm)

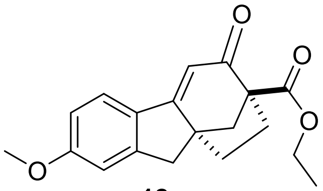

12

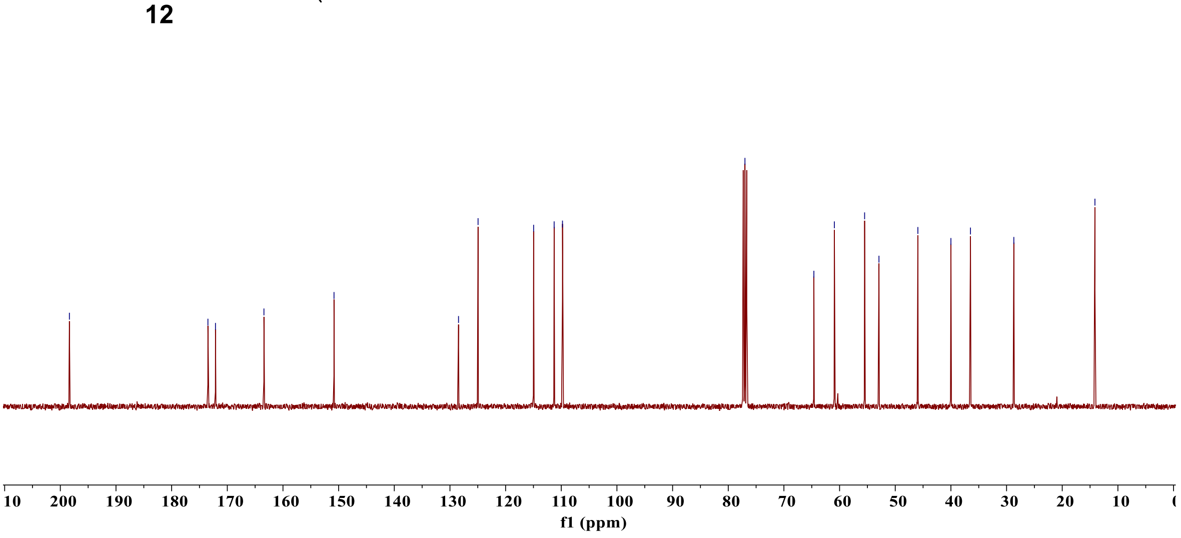
198.37
173.48
172.13
163.42
150.83
128.46
124.95
114.97
111.28
109.78
77.00
64.62
60.94
55.51
52.92
45.93
40.00
36.48
28.70
14.13
200 190 180 170 160 150 140 130 120 110 100 90 80 70 60 50 40 30 20 10
f1 (ppm)

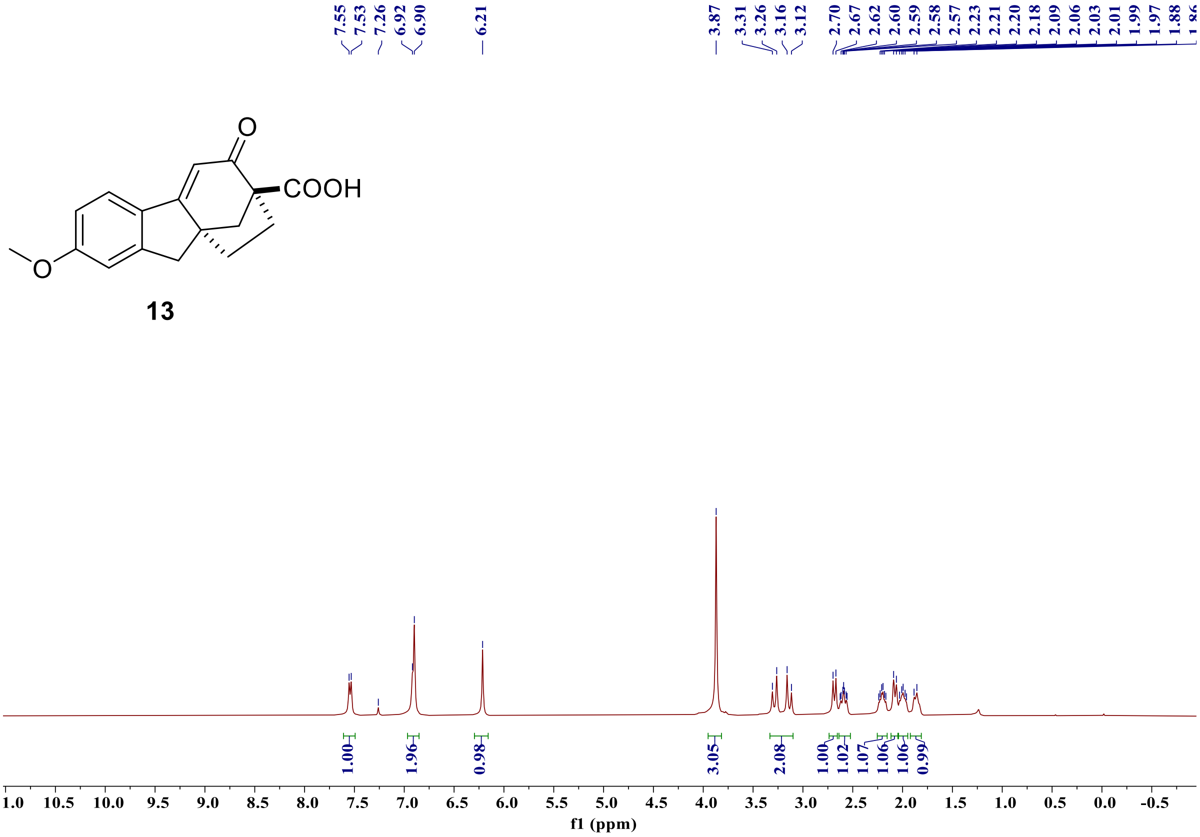
7.55
7.53
7.26
6.92
6.90
6.21
3.87
3.31
3.26
3.16
3.12
2.70
2.67
2.62
2.60
2.59
2.58
2.57
2.23
2.21
2.20
2.18
2.09
2.06
2.03
2.01
1.99
1.97
1.88
O
COOH
O
13
1.00
1.96
0.98
3.05
2.08
1.00
1.02
1.07
1.06
1.06
0.99
f1 (ppm)

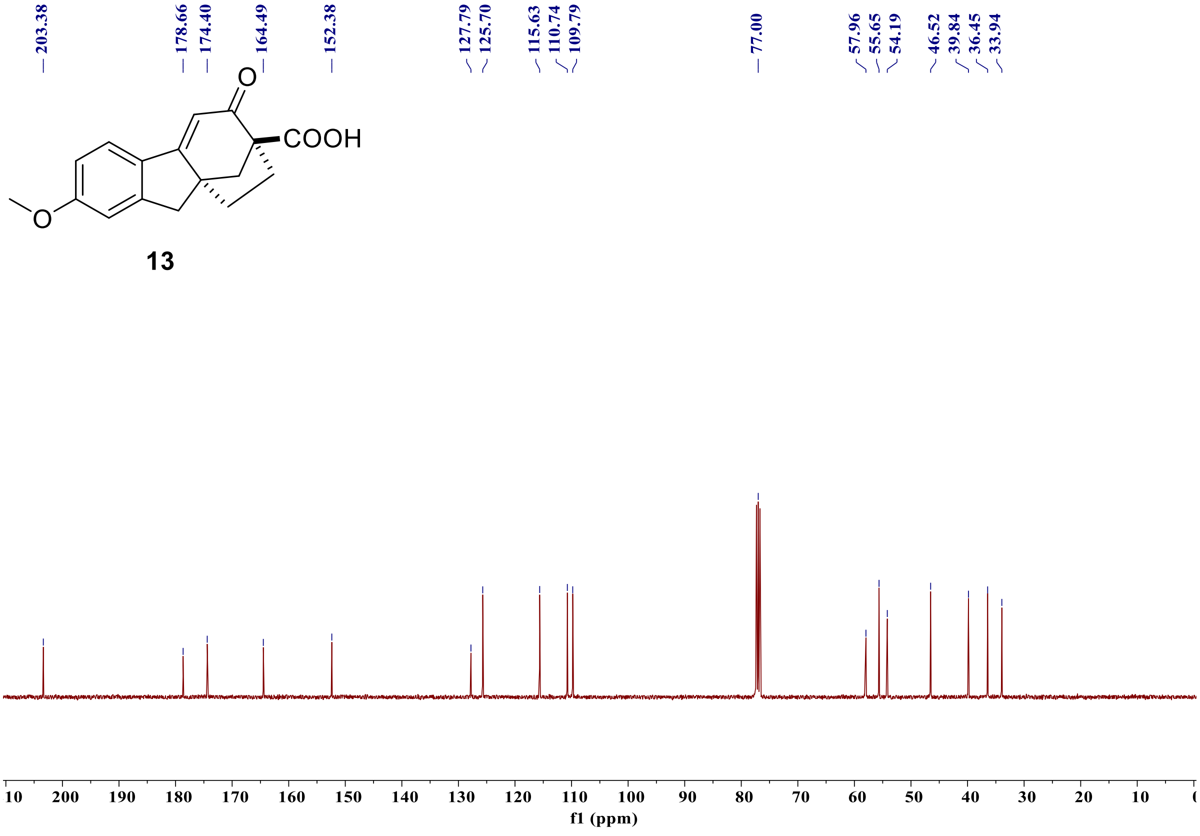
203.38
178.66
174.40
164.49
152.38
127.79
125.70
115.63
110.74
109.79
77.00
57.96
55.65
54.19
46.52
39.84
36.45
33.94
O
COOH
O
13
f1 (ppm)

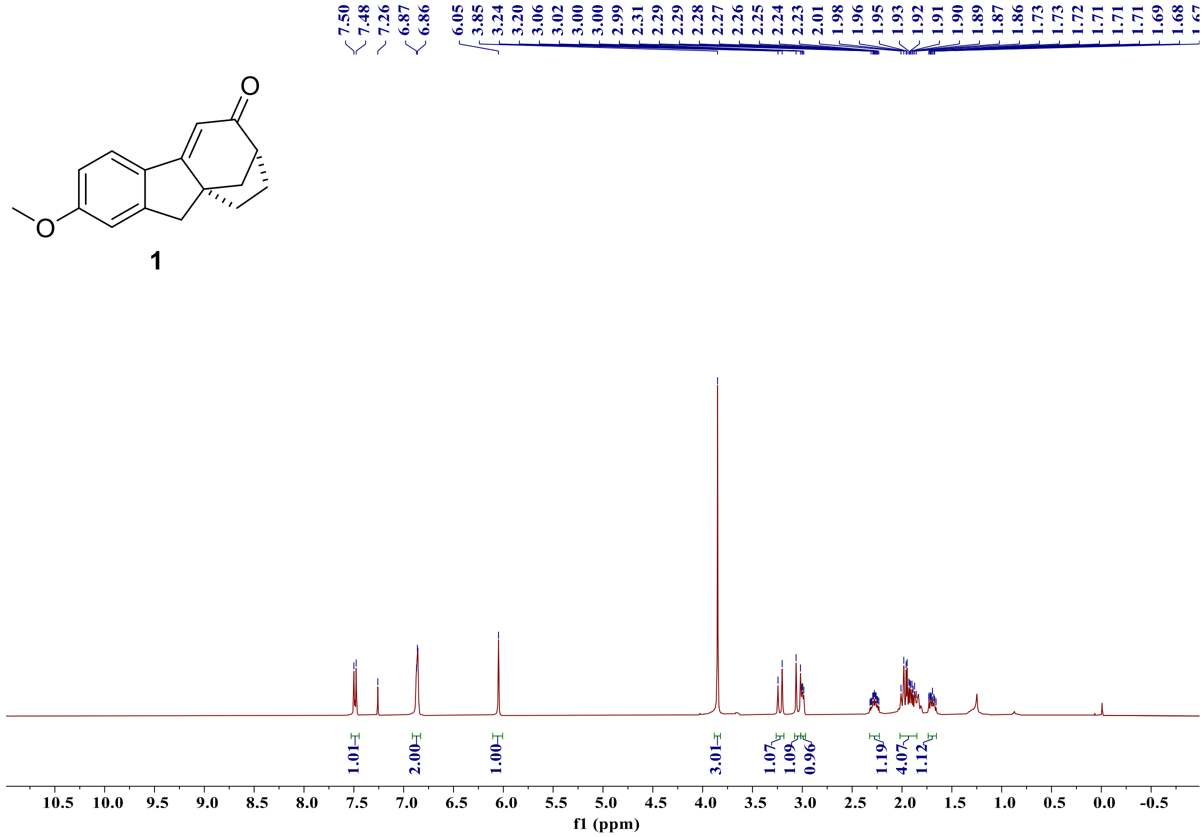
7.50
7.48
7.26
6.87
6.86
6.05
3.85
3.24
3.20
3.06
3.02
3.00
3.00
2.99
2.31
2.29
2.29
2.28
2.27
2.26
2.25
2.24
2.23
2.01
1.98
1.96
1.95
1.93
1.92
1.91
1.90
1.89
1.87
1.86
1.73
1.73
1.72
1.71
1.71
1.71
1.69
1.68
1
1.01
2.00
1.00
3.01
1.07
1.09
0.96
1.19
4.07
1.12
f1 (ppm)

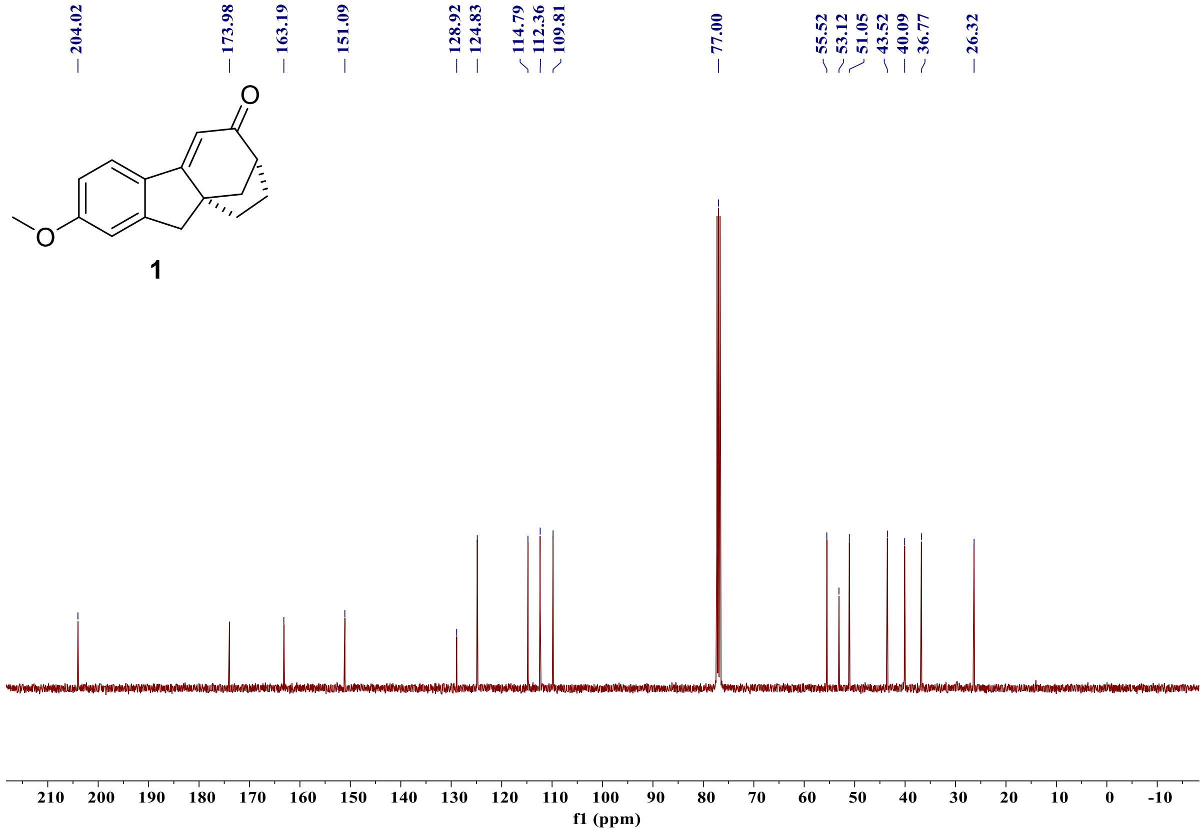
204.02
173.98
163.19
151.09
128.92
124.83
114.79
112.36
109.81
77.00
55.52
53.12
51.05
43.52
40.09
36.77
26.32
1
f1 (ppm)
